# Efficacy and safety of interventions to control myopia progression in children: an overview of systematic reviews and meta-analyses

**DOI:** 10.1186/s12886-019-1112-3

**Published:** 2019-05-09

**Authors:** Efthymia Prousali, Anna-Bettina Haidich, Andreas Fontalis, Nikolaos Ziakas, Periklis Brazitikos, Asimina Mataftsi

**Affiliations:** 1 2nd Department of Ophthalmology, Aristotle University of Thessaloniki, Thessaloniki, Greece; 2 Department of Hygiene, Social-Preventive Medicine and Medical Statistics, Aristotle University of Thessaloniki, Thessaloniki, Greece; 3 Sheffield Teaching Hospitals NHS Foundation Trust, Northern General Hospital, Herries Rd, Sheffield, UK

**Keywords:** Refractive error, Myopia, Children, Vision, Lenses, Anti-muscarinic agents

## Abstract

**Background:**

Myopia is a common visual disorder with increasing prevalence. Halting progression of myopia is critical, as high myopia can be complicated by a number of vision-compromising conditions.

**Methods:**

Literature search was conducted in the following databases: Medical Literature Analysis and Retrieval System Online (MEDLINE), Excerpta Medica dataBASE (EMBASE), Cochrane Database of Systematic Reviews (CDSR), Database of Abstracts of Reviews of Effects (DARE) and Centre for Reviews and Dissemination (CRD) Health Technology Assessment (HTA) database. Systematic reviews and meta-analyses investigating the efficacy and safety of multiple myopia interventions vs control conditions, were considered. Methodological quality and quality of evidence of eligible studies were assessed using the ROBIS tool and GRADE rating. The degree of overlapping of index publications in the eligible reviews was calculated with the corrected covered area (CCA).

**Results:**

Forty-four unique primary studies contained in 18 eligible reviews and involving 6400 children were included in the analysis. CCA was estimated as 6.2% and thus considered moderate. Results demonstrated the superior efficacy of atropine eyedrops; 1% atropine vs placebo (change in refraction: -0.78D, [− 1.30 to − 0.25] in 1 year), 0.025 to 0.05% atropine vs control (change in refraction: -0.51D, [− 0.60 to − 0.41] in 1 year), 0.01% atropine vs control (change in refraction: -0.50D, [− 0.76 to − 0.24] in 1 year). Atropine was followed by orthokeratology (axial elongation: − 0.19 mm, [− 0.21 to − 0.16] in 1 year) and novel multifocal soft contact lenses (change in refraction: -0.15D, [− 0.27 to − 0.03] in 1 year). As regards adverse events, 1% atropine induced blurred near vision (odds ratio [OR] 9.47, [1.17 to 76.78]) and hypersensitivity reactions (OR 8.91, [1.04 to 76.03]).

**Conclusions:**

Existing evidence has failed to convince doctors to uniformly embrace treatments for myopic progression control, possibly due to existence of some heterogeneity, reporting of side effects and lack of long-term follow-up. Research geared towards efficient interventions is still necessary.

**Electronic supplementary material:**

The online version of this article (10.1186/s12886-019-1112-3) contains supplementary material, which is available to authorized users.

## Background

Myopia is a common condition exhibiting an “epidemic” during the past half-century. East and Southeast Asia appear to have a higher myopia prevalence, compared to Western and European populations, with Singapore, China, Hong Kong and Taiwan representing the regions where the problem is more common [1]. Myopia is included in the 10 priority eye diseases in VISION 2020 campaign for the prevention of blindness and visual impairment, as declared by the World Health Organization [2].

Myopia introduces significant social and psychological impact, as it appears to affect children’s perception of their physical appearance, athletic competence and social acceptance [3]. Myopia also imposes a considerable economic burden to societies. Annual expenses for myopia treatment are estimated to be greater than for other ocular diseases including age-related macular degeneration and primary open-angle glaucoma, as well as for non-ocular chronic pathologies, such as Parkinson’s disease and chronic obstructive pulmonary disease [4]. A treatment that would halt or at least decelerate myopia’s progression rate is highly desirable, as severe myopia constitutes a substantial risk factor for several ocular conditions which can lead to blindness. These include retinal detachment, primary open angle glaucoma, cataract and macular degeneration [1].

Several interventions have been attempted to control myopic progression, some of which showed no effect and others were effective but with limitations [5–8]. Long-term safety and efficacy of interventions to restrict myopia remains unresolved, resulting in the lack of universal consensus in myopia treatment [9–12]. As there appears to be no overview in existing literature, the aim of the present study is to synthesize evidence provided by systematic reviews (SRs) and meta-analyses (MAs) on myopia control.

## Methods

### Protocol and registration

We used the term ‘overview’ for our synthesis of multiple intervention systematic reviews and meta-analyses, as proposed by the Cochrane Collaboration [13] and reporting followed the PRIO-harms guidelines (Additional file 1: Appendix 1) [14]. The protocol of this study is registered in the PROSPERO database (CRD42017068204) [15] and published in *Systematic reviews* [15]*.*

### Ιnformation sources and search strategy

Purposive literature search was conducted in the Cochrane Database of Systematic Reviews (CDSR), Database of Abstracts of Reviews of Effects (DARE) and Centre for Reviews and Dissemination (CRD) Health Technology Assessment (HTA) Database, using the keyword ‘myopia’. A more comprehensive search strategy was applied in MEDLINE and EMBASE, using medical subject headings (MeSH) and text words related to spectacles, contact lenses, anti-muscarinic agents, myopia and children [5, 16] to find any recent primary studies not included in the published systematic reviews. The last search date was March 9, 2018. For all included studies, reference lists were also searched. MEDLINE search strategy is provided (Additional file 1: Appendix 2). No language, study type or date restrictions were used.

### Eligibility criteria

#### Participants

Our overview target were children and adolescents, ≤ 18 years of age at baseline, diagnosed with myopia defined as spherical equivalent refraction ≤ − 0.25 dioptres, with or without astigmatism, without any ocular comorbidities including strabismus and amblyopia. Animals, adult population, patients not suffering from myopia, or patients with myopia and strabismus/amblyopia were excluded. Studies related to surgical interventions for myopia correction, e.g. refractive surgery were not considered.

#### Interventions and comparators

We included studies in which any optical or pharmacological intervention for myopia control was compared to single vision spectacles, contact lenses, or placebo. No restriction on duration and dose of treatment, if applicable, was imposed.

#### Outcome measures

Our primary outcomes regarded myopia progression and axial elongation as efficacy criteria. Myopia progression was assessed as mean change in refractive error, measured in dioptres. Mean change in axial length, measured in millimetres, was also evaluated. Outcomes reporting change in a 12-month or 24-month period were accepted and described. Reported adverse events (AE) were regarded as safety criteria.

#### Study design

Unit of analysis of this overview were SRs or meta-analyses of randomized controlled trials (RCTs), pseudo-RCTs, cohort and case-control studies. Network meta-analyses were also reviewed. Only human studies with full text available were analysed. Narrative reviews that do not systematically search the literature and do not critically appraise the quality of included studies were excluded.

Subsequently, primary studies included in eligible systematic reviews and meta-analyses were identified and employed as a unit of analysis to perform an extensive meta-analysis and provide effect estimates for myopia control interventions (thereafter referred to as index publications). The total of index publications included in the meta-analysis is provided in Additional file 1: Appendix 3. Levels of evidence as produced by the Oxford Centre for Evidence Based Medicine (OCEBM) were considered [17]. Index publications of low level of evidence, i.e. poor quality cohort/case-control studies, case series, case reports or expert opinions were not included. Cohort and case-control studies were considered of low quality if they scored less than 5 points in Newcastle-Ottawa scale. Similarly, index publications without a control group or those comparing two or more different interventions were not included in the statistical analysis.

### Study selection and data management

Two independent authors (EP, AF) performed all screening steps. Title and abstract screening were conducted using the Mendeley citation management software. The overview authors screened the titles and abstracts against the eligibility criteria and obtained full reports for all titles that appeared to meet the inclusion criteria or where there was uncertainty. The same two independent authors (EP, AF) managed data in duplicate from each eligible study, using a data collection form in Microsoft Excel designed to include all the data required. Each SR or MA was initially evaluated to identify whether it matched the eligibility criteria of the overview. Subsequently, index publications contained in these SRs/MAs were individually reviewed. Data extraction was then performed for inclusion of eligible index publications in the meta-analysis. In cases where risk of bias of index publications was not available by the included SR or MA, two independent authors (EP, AF) completed the missing assessments. When an index publication was included in more than one SRs or MAs, outcome data were extracted from the most comprehensive study. A third author was involved to resolve any discrepancies, using the primary research paper (ABH).

### Risk of bias assessment

Two overview authors (EP, AF) independently assessed the methodological quality of each included SR and MA using the Risk Of Bias In Systematic Reviews (ROBIS) tool [18]. The quality of evidence was evaluated by two independent authors (EP, AF) using four domains of the Grading of Recommendations Assessment, Development, and Evaluation (GRADE) tool: study limitations, imprecision, inconsistency of results, and indirectness and a summary of findings for each outcome of interest was designed using GRADEpro software [19, 20]. In order to minimize the subjectivity of quality assessment process, a third reviewer was involved to resolve any discrepancies (ABH).

The list of included index publications in eligible SRs/MAs was reviewed in order to identify those contained in two or more reviews. We generated a citation matrix presenting all the SRs/MAs in columns and all included index publications in rows. We estimated the overlap by calculating the corrected covered area (CCA), to assess if specific index publications are overrepresented. The formula for calculating CCA is: **CCA =** $\frac{\mathrm{N}-\mathrm{r}}{\mathrm{rc}-\mathrm{r}}$ where N = sum of the included studies, r = rows (number of unique studies), c = columns (number of reviews). CCA reflected the degree of actual overlap, as it is not influenced by large reviews. Should high or very high overlap be detected, which is interpreted as CCA equal to or more than 10%, we planned to retain the review which is (1) the most recent, (2) containing a higher amount of information, and (3) the most rigorous in terms of methodology, as assessed by ROBIS tool and GRADE scale [21, 22]. In addition, two independent overview authors (EP, AF) examined possible presence of meta-biases, including publication bias, selective outcome reporting and dual co-authorship. Handling of inconsistency for meta-analyses and other potential sources of bias are also reported (Additional file 1: Table S1) [23–26].

### Data synthesis and analysis

Index publications included in eligible systematic reviews and meta-analyses were employed as a unit of analysis to perform a meta-analysis using Review Manager software version 5.3. Continuous outcomes were expressed using mean differences with 95% confidence intervals (CIs) and dichotomous outcomes were expressed using odd ratios (ORs) with 95% CIs. Data were synthesized using random-effects models due to the inconsistency across the RCTs and cohort studies. Subgroup analyses according to study design (RCTs and observational studies) were performed and whenever no subgroup differences were obtained the overall effect was reported. We pooled the results referring to one eye only, to each eye separately, or to the average of both eyes, depending on the data analysed by each index publication design (Additional file 1: Table S2). Sensitivity analyses excluding studies of lower methodological quality, when each eye was reported separately or those introducing substantial inconsistency were also conducted.

## Results

**Fig. 1:**
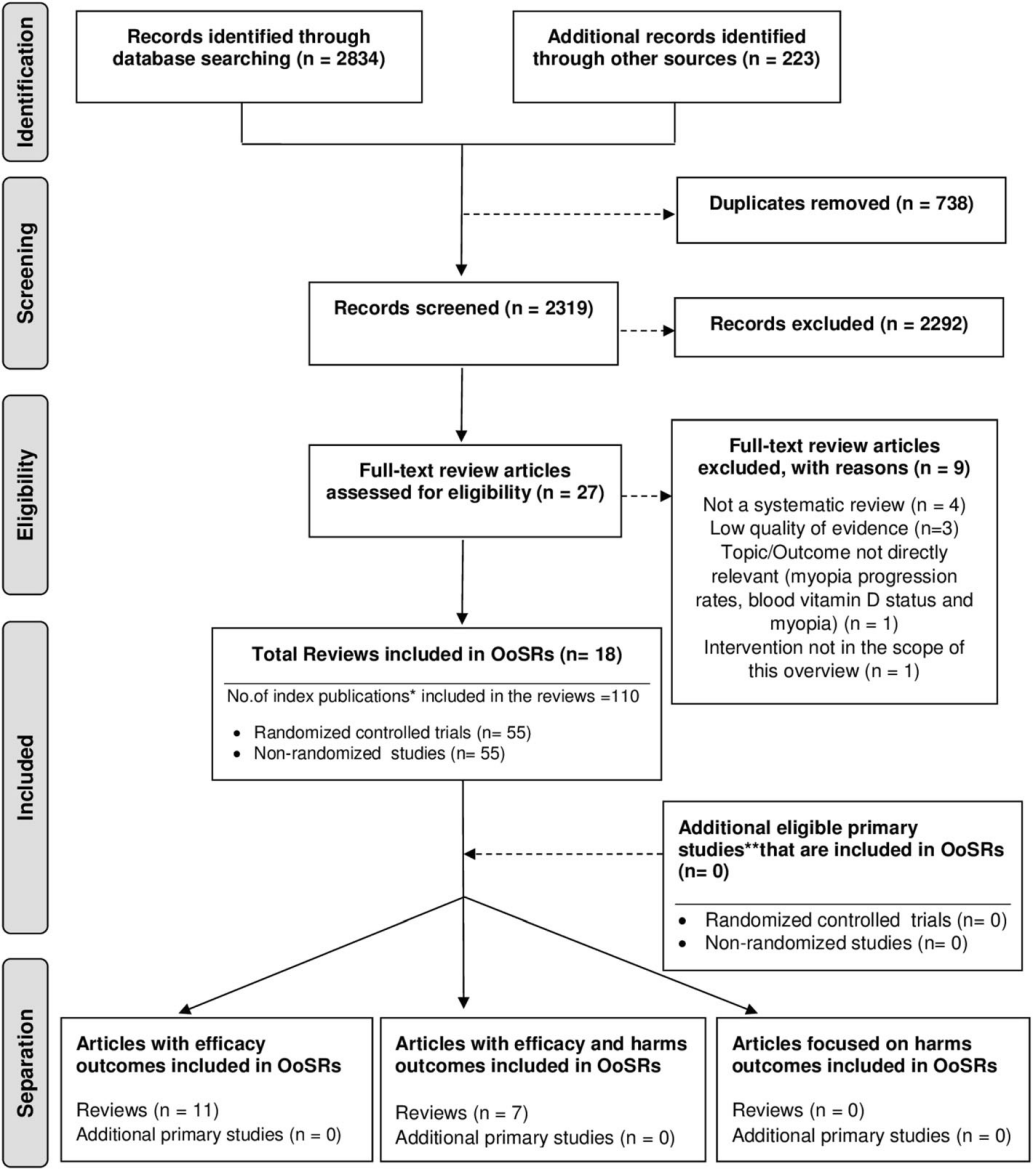
Flow chart for Overview of Systematic Reviews (OoSRs). *Index publication is the first occurrence of a primary publication in the included reviews. **Additional eligible primary studies that had not been initially identified by the search of the relevant reviews or obtained by updating the search of the included reviews

Literature search yielded 3057 records, 2319 non-duplicates were screened and 27 retrieved in full text (Fig. 1) [14]. Nine studies were subsequently excluded (Additional file 1: Table S3), leaving 18 SRs and meta-analyses to be included in this overview [5, 6, 9–12, 27–38]**.**

Table 1 summarizes the main characteristics of the 18 eligible SRs/MAs. These were published between 2002 and 2017. Five studies are SRs and meta-analyses, four performed systematic review of literature with qualitative syntheses of findings, eight performed meta-analyses, and one is a network meta-analysis. Four studies investigated atropine, four analysed orthokeratology, two focused on outdoors exposure, one examined the efficacy of acupuncture, and two investigated the use of multifocal lenses. The remaining five studies examined multiple interventions for myopia control.

**Table 1:** Characteristics of included studies

Review &Type of Study	Databases searched and last assessed	No. of primary studies (sample size)	Ethnicity	Age range (average age) in years	Treatment	Control	Overview outcomes
Walline et al. 2011 [5] SR & meta-analysis	CENTRAL, MEDLINE, EMBASE, LILACS, *m*RCT, ClinicalTrials.gov (10/2011)	23 (4696)	Israel, Malaysia, China, USA, Finland, Hong Kong, Japan, Taiwan, Denmark	6–18	Undercorrection, multifocal spectacles, bifocal soft contact lenses, novel lenses, RGPCLs, anti-muscarinic medications	Full correction spectacles, SVLs, single vision contact lenses, placebo	Change in RE, change in AL
Sherwin et al. 2012 [36] SR & meta-analysis	MEDLINE, EMBASE, Web of Science, CENTRAL (NA)	23 (80–3009)	Singapore, Australia, Jordan, China, USA	0.5–20	Outdoor exposure	NA	Risk for myopia progression
Wen et al. 2015 [33] SR & meta-analysis	MEDLINE, EMBASE, Cochrane Library, WHO international Clinical Trials Registry Platform, ClinicalTrials.gov (11/2014)	8 (769)	Chinese, Caucasian, Japanese	6–15	OK	Single vision spectacles, contact lenses	Change in AL
Xiong et al. 2017 [12] SR & meta-analysis	PubMed, EMBASE, Cochrane Library (12/2015)	25 (50–5048)	China, Taiwan, Singapore, Australia, UK, USA, Turkey	6–18^a^	Outdoor exposure	NA	Risk of myopia progression
Gong et al. 2017 [9] SR & meta-analysis	PubMed, EMBASE, Cochrane Central Register of Controlled Trials (04/2016)	19 (3137)	Taiwan, USA, Singapore, China, Hong Kong	5–17	Atropine	Atropine, control conditions	Myopia progression, adverse events
Saw et al. 2002 [6] SR	MEDLINE, EMBASE, Cochrane Library, Science Citation Index (2000)	26 (32–247)	Taiwan, USA, Finland, Denmark	NA	Atropine, timolol, bifocals, multifocal lenses, multifocal lenses + atropine, contact lenses	Tropicamide, cyclopentolate, lenses, bifocals, single vision spectacles	Change in RE
Wei et al. 2011 [27] SR	CENTRAL, MEDLINE, EMBASE, AMED, LILACS, *m*RCT, ClinicalTrials.gov, NCCAM, CBM, CNKI, VIP (07/2011)	2 (131)	Taiwan	< 18	Auricular stimulation with 0.25% atropine, acupressure and interactive multimedia	Placebo, sham acupuncture, atropine eyedrops 0.25% or 0.5%, non-specific treatment, e.g. vitamin E, spectacles	Change in RE, change in AL
Chassine et al. 2015 [38] SR	MEDLINE, Google Scholar (12/2014)	19 (26–1209)	Αustralia, Singapore, China, USA, Malaysia, Canada, New Zealand, Russia	NA	Outdoor activity, atropine, undercorrection, bifocal (prismatic) spectacles, CLs, multifocal SCLs, OK, RGPCLs	Full correction, SVLs, atropine, PALs, SCLs	Myopia progression
Shih et al. 2016 [28] SR	US FDA website, PubMed, ClinicalTrials.gov, Cochrane Library (04/2015)	5 (96–400)	Taiwan, Singapore	6–13	Atropine 0.1–1%	Saline, cyclopentolate, atropine, tropicamide, atropine + multifocal spectacles, multifocal spectacles, SVLs	Change in RE, adverse events
Song et al. 2011 [29] Meta-analysis	Cochrane Library, PubMed, US FDA website, ClinicalTrials.gov, European regulatory authorities, manufacturer’s product information sheets, CBMDISC (2009)	6 (823)	Taiwan, Hong Kong, Singapore	5–15	Atropine 0.1–1%	Atropine, atropine + multifocal spectacles, multifocal spectacles/SVLs, auricular acupoints, tropicamide, cyclopentolate	Change in RE, change in AL
Li et al. 2011 [30] Meta-analysis	MEDLINE, EMBASE, Cochrane Library, Science Citation Index, Chinese Clinical Trial Registry, WHO international Clinical Trials Registry Platform, ClinicalTrials.gov (10/2010)	9 (1464)	USA, Hong Kong, China, Taiwan, Japan, Canada, Finland	6–13	Multifocal lenses (bifocal lenses, PALs)	SVLs	Change in RE, change in AL
Li et al. 2014 [37] Meta-analysis	MEDLINE, EMBASE, Cochrane Library, WHO international Clinical Trials Registry Platform, ClinicalTrials.gov (04/2013)	11 (1815)	Taiwan, Hong Kong, Singapore, United States	8–15	Atropine 0.025–1%	Placebo, Tropicamide, blank	Change in RE
Sun et al. 2015 [31] Meta-analysis	MEDLINE, EMBASE (01/2014)	7 (435)	Hong Kong, Japan, Spain, USA	6–16	OK	Spectacles, SCLs	Change in AL
Si et al. 2015 [32] Meta-analysis	PubMed, EMBASE, Cochrane Library (11/2013)	7 (435)	Hong Kong, Japan, Spain, USA	6–16	OK	SVLs, spectacles, SCLs	Change in AL
Li et al. 2016 [10] Meta-analysis	MEDLINE, EMBASE, Cochrane Library, Chinese Clinical Trial Registry, WHO international Clinical Trials Registry Platform, ClinicalTrials.gov (01/2015)	9 (667)	Hong Kong, Japan, China, Spain	6–16	OK	SVLs	Change in AL, adverse events
Li et al. 2017 [11] Meta-analysis	MEDLINE, EMBASE, Cochrane Library, Chinese Clinical Trial Registry, WHO international Clinical Trials Registry Platform, ClinicalTrials.gov (05/2016)	8 (587)	USA, China, Hong Kong, New Zealand, Japan, Spain	6–18	SCLs with concentric ring bifocal and peripheral add multifocal designs	single vision SCLs or spectacles	Change in RE, change in AL
Cui et al. 2017 [35] Meta-analysis	MEDLINE, Cochrane, EMBASE, Google Scholar (09/2015)	5 (673)	USA, Singapore, East Asia	6–16	RGPCLs	SCLs, spectacles, OK	Change in RE, change in AL
Huang et al. 2016 [34] Network meta-analysis	MEDLINE, EMBASE, Cochrane Central Register of Controlled Trials, WHO international Clinical Trials Registry Platform, ClinicalTrials.gov (08/2014)	30 (5387)	Israel, Malaysia, Hong Kong, USA, Denmark, Finland, Japan, China, New England, Singapore, Taiwan	< 18	Atropine, pirenzepine, timolol, cyclopentolate, SVLs, OK, RGPCLs, bifocal spectacles outdoor activity, PALs, prismatic bifocals, peripheral defocus modifying spectacles and lenses, SCLs	Tropicamide, SVLs	Change in RE, change in AL

*AL* axial length, *AMED* Allied and Complementary Medicine Database, *CBM* Chinese Biological Medicine Database, *CBMDISC* Chinese Biomedical Literature Analysis and Retrieval System for Compact Disc, *CLs* contact lenses, *CNKI* China National Knowledge Infrastructure, *FDA* Food and Drug Administration, *LILACS* Latin American and Caribbean Health Sciences Literature Database, *mRCT meta*Register of Controlled Trials, *NA* not available, *NCCAM* National Center for Complementary and Alternative Medicine, *No.* number, *OK* orthokeratology, *RE* refractive error, *RCTs* randomized controlled trials, *RGPCLs* rigid gas permeable contact lenses, *PALs* progressive addition lenses, *SCLs* soft contact lenses, *SVLs* single vision lenses, *SVSCLs* single vision soft contact lenses, *SR* systematic review, *WHO* World Health Organization

^a^Age range refers to the majority of participants

### Overlapping

The 18 included SRs and MAs comprised 226 overlapping index publications, of which 110 were unique. Two recently published RCTs not included in the 18 SRs/MAs were also identified through literature search and added to the index publications. A citation matrix presenting all the included SRs/MAs in columns and index publications in rows is provided in Additional file 1: Table S4. Index publications represented in more than one eligible reviews are recognized in the citation matrix. In order to avoid potential double counting of outcomes, we calculated the degree of actual overlap by estimating the CCA:

$$ \mathbf{CCA}=\frac{\mathrm{N}-\mathrm{r}}{\mathrm{rc}-\mathrm{r}}=\frac{226-110}{110\times 18-110}=\frac{116}{1870}=\sim 6.2\% $$

As CCA is estimated at 6.2%, the overlap is in the moderate range reflecting a moderate risk of skewed reporting [21, 22]. Out of the 112 index publications, 44 matched our eligibility criteria and were included in the meta-analysis. These consisted of 28 RCTs and 16 observational studies, and reported data on a total of 6400 patients.

### Assessment of methodological quality

Qualitative, domain-based rating of methodological quality of eligible studies with ROBIS tool is provided in Additional file 1: Table S5 [18]. The overall risk of bias was ‘low’ in 14 reviews [5, 6, 9–11, 27–35]**,** ‘unclear’ in three [12, 36, 37] and ‘high’ in one review [38].

With regard to ‘Study eligibility criteria’ domain, two studies [6, 38] were judged with ‘high concern’ due to imprecisely defined eligibility criteria, publication status and/or language limitations. One study [38] was judged with ‘high’ and one [29] with ‘unclear concern’ in ‘Identification and selection of studies’ domain because of limited details regarding search strategy and unclear study selection process. Domain 3 assessed the methodology used for data collection and study appraisal, in which two studies [6, 28] were judged with ‘unclear’ and one [38] with ‘high concern’, due to lack of information on included studies’ characteristics for appropriate interpretation of findings, or because of inappropriate or no risk of bias assessment of index publications. Fifteen eligible reviews reported quality assessment of the included index publications. Four [29–32] used the Jadad scale and four [10, 11, 33, 37] combined Jadad with Newcastle Ottawa scale for observational studies, four [5, 27, 34, 35] used the Cochrane Collaboration Risk of Bias tool, one [9] combined the Cochrane tool with Newcastle Ottawa scale and two [12, 36] used other tools. Three reviews [6, 28, 38] did not report formal assessment of included index publications. In ‘Synthesis and findings’ domain, three studies [36–38] were judged with ‘high’ and one [12] with ‘unclear concern’, due to inappropriate quantitative or qualitative synthesis.

None of the eligible studies reported a GRADE assessment. Two authors (EP, AF) independently rated the quality of evidence for our outcomes using the GRADE scale (Tables 2, 3, 4, 5 and 6), [19–21]. Quality was assessed as ‘high’ in one outcome, ‘moderate’ in 41, ‘low’ in 12 and ‘very low’ in 23 outcomes examining efficacy or safety (Additional file 1: Appendix 6). Low/very low quality is due to a number of index publications being at risk of bias from elements involving imprecision, inconsistency and limitations including lack of blinding or allocation concealment and loss to follow-up.

**Table 2:** Primary outcomes from baseline (1 year) - Change in refractive error

Outcome	Comparison	Number of subjects (primary studies)	Measure of effect (95% CI)	Direction of effect	I^2^ (%)
Change in refractive error	Undercorrected versus fully corrected spectacles	142 (2)	MD = 0.15 (0.00 to 0.29)	Favours fully corrected spectacles	0
	Bifocal spectacles versus SVLs	259 (2)	MD = − 0.09 (− 0.19 to 0.02)	Favours bifocal spectacles	0
	1% atropine versus placebo - RCTs	604 (3)	MD = − 0.78 (− 1.30 to − 0.25)	Favours atropine	97
	1% atropine versus control - cohort studies	798 (3)	MD = − 0.39 (− 0.59 to − 0.19)	Favours atropine	26
	0.025 and 0.05% atropine versus control	224 (3)	MD = − 0.51 (− 0.60 to − 0.41)	Favours atropine	9
	0.01% atropine versus control	60 (1)	MD = − 0.50 (− 0.76 to − 0.24)	Favours atropine	N/A
	2% pirenzepine gel versus placebo	84 (1)	MD = − 0.30 (− 0.51 to − 0.09)	Favours pirenzepine	N/A
	RGPCLs versus spectacles or SCLs	420 (2)	MD = − 0.08 (− 0.19 to 0.02)	Favours RGPCLs	91
	Concentric ring bifocal SCLs versus SVSCLs	264 (3)	MD = − 0.31 (− 0.60 to 0.02)	Favours concentric ring bifocal SCLs	88
	Peripheral add multifocal SCLs versus SVLs - RCTs	294 (5)	MD = − 0.23 (− 0.31 to − 0.14)	Favours peripheral add multifocal SCLs	0
	ΟΚ versus SCLs or SVLs	39 (1)	MD = − 0.27 (− 0.50 to − 0.04)	Favours OK	N/A
	PALs versus SVLs	206 (2)	MD = − 0.10 (− 0.21 to 0.00)	Favours PALs	0

*CI* confidence interval, *MD* Mean Difference, *N/A* not applicable, *PALs* progressive addition lenses, *RCTs* randomized controlled trials, *RGPCLs* rigid gas permeable contact lenses, *SCLs* soft contact lenses, *SVLs* single vision lenses, *SVSCLs* single vision soft contact lenses

**Table 3:** Primary outcomes from baseline (1 year) – Change in axial length

Outcome	Comparison	Number of subjects (primary studies)	Measure of effect (95% CI)	Direction of effect	I^2^ (%)
Change in axial length	Undercorrected versus fully corrected spectacles	94 (1)	MD = 0.05 (− 0.01 to 0.11)	Favours full correction	N/A
	RGPCLs versus spectacles or SCLs	415 (2)	MD = − 0.02 (− 0.05 to 0.10)	Favours spectacles/SCLs	0
	2% pirenzepine gel versus placebo	264 (2)	MD = − 0.10 (− 0.18 to − 0.01)	Favours pirenzepine	0
	Concentric ring bifocal SCLs versus SVSCLs	264 (3)	MD = − 0.12 (− 0.19 to − 0.06)	Favours concentric ring bifocal SCLs	66
	1% atropine versus control	586 (3)	MD = − 0.36 (− 0.41 to − 0.30)	Favours atropine	46
	Peripheral add multifocal SCLs versus SVLs - RCTs	294 (5)	MD = − 0.10 (− 0.14 to − 0.05)	Favours peripheral add multifocal SCLs	37
	ΟΚ versus SCLs or SVLs	524 (8)	MD = − 0.19 (− 0.21 to − 0.16)	Favours OK	0
	PALs versus SVLs	211 (2)	MD = − 0.08 (− 0.14 to 0.02)	Favours PALs	65

*CI* confidence interval, *MD* Mean Difference, *N/A* not applicable, *OK* Orthokeratology, *PALs* progressive addition lenses, *RGPCLs* rigid gas permeable contact lenses, *SCLs* soft contact lenses, *SVLs* single vision lenses, *SVSCLs* single vision soft contact lenses

**Table 4:** Primary outcomes from baseline (2 years) – Change in refractive error

Outcome	Comparison	Number of subjects (primary studies)	Measure of effect (95% CI)	Direction of effect	I^2^ (%)
Change in refractive error	Undercorrected versus fully corrected spectacles	142 (2)	MD = 0.17 (0.12 to 0.23)	Favours fully corrected spectacles	0
	Bifocal spectacles versus single vision lens spectacles	351 (3)	MD = − 0.19 (− 0.59 to 0.21)	Favours bifocal spectacles	85
	1% atropine versus placebo	400 (1)	MD = − 0.92 (− 1.08 to − 0.76)	Favours atropine	N/A
	2% pirenzepine gel versus placebo	74 (1)	MD = − 0.41 (− 0.70 to − 0.12)	Favours pirenzepine	N/A
	RGPCLs versus spectacles or SCLs	398 (2)	MD = − 0.16 (− 0.33 to − 0.00)	Favours RGPCLs	92
	Concentric ring bifocal SCLs versus SVSCLs	128 (1)	MD = − 0.20 (− 0.38 to − 0.02)	Favours concentric ring bifocal SCLs	N/A
	Peripheral add multifocal SCLs versus SVLs	99 (2)	MD = − 0.50 (− 0.65 to − 0.35)	Favours peripheral add multifocal SCLs	0
	ΟΚ versus SCLs or SVLs	39 (1)	MD = − 0.66 (−1.01 to − 0.31)	Favours OK	N/A
	PALs versus SVLs	940 (4)	MD = − 0.15 (− 0.40 to 0.11)	Favours PALs	89

*CI* confidence interval, *MD* Mean Difference, *N/A* not applicable, *PALs* progressive addition lenses, *RGPCLs* rigid gas permeable contact lenses, *SCLs* soft contact lenses, *SVLs* single vision lenses, *SVSCLs* single vision soft contact lenses

**Table 5:** Primary outcomes from baseline (2 years) – Change in axial length

Outcome	Comparison	Number of subjects (primary studies)	Measure of effect (95% CI)	Direction of effect	I^2^ (%)
Change in axial length	Undercorrected versus fully corrected spectacles	94 (1)	MD = 0.06 (− 0.04 to 0.16)	Favours full correction	N/A
	Bifocal spectacles versus single vision lens spectacles	89 (1)	MD = − 0.20 (− 0.31 to − 0.09)	Favours bifocal spectacles	N/A
	1% atropine versus placebo	400 (1)	MD = − 0.36 (− 0.43 to − 0.29)	Favours atropine	N/A
	2% pirenzepine gel versus placebo	74 (1)	MD = − 0.12 (− 0.29 to 0.05)	Favours pirenzepine	N/A
	RGPCLs versus spectacles or SCLs	394 (2)	MD = 0.03 (− 0.05 to 0.12)	Favours spectacles or SCLs	0
	Concentric ring bifocal SCLs versus SVSCLs	128 (1)	MD = − 0.12 (− 0.20 to − 0.04)	Favours concentric ring bifocal SCLs	N/A
	Peripheral add multifocal SCLs versus SVLs	99 (2)	MD = − 0.13 (− 0.20 to − 0.06)	Favours peripheral add multifocal SCLs	0
	ΟΚ versus SCLs or SVLs	663 (11)	MD = − 0.27 (− 0.31 to − 0.23)	Favours OK	0
	PALs versus SVLs	791 (3)	MD = −0.10 (− 0.20 to 0.00)	Favours PALs	78

*CI* confidence interval, *N/A* not applicable, *OK* Orthokeratology, *PALs* progressive addition lenses, *RGPCLs* rigid gas permeable contact lenses, *SCLs* soft contact lenses, *SVLs* single vision lenses, *SVSCLs* single vision soft contact lenses

**Table 6:** Primary outcomes from baseline – Adverse Events

Outcome	Comparison	Number of subjects (primary studies)	Measure of effect (95% CI)	Direction of effect	I^2^ (%)
Allergic or hypersensitivity reactions or discomfort	1% atropine versus control	446 (2)	OR = 8.91 (1.04, 76.03)	Favours control	0
Blurred near vision	1% atropine versus control	540 (2)	OR = 9.47 (1.17, 76.78)	Favours control	0
Contact lens-related discomfort/Unwillingness to wear contact lenses	Concentric ring bifocal SCLs versus SVSCLs	261 (2)	OR = 0.95 (0.49, 1.81)	Favours concentric ring bifocal SCLs	0
Mild corneal erosion	ΟΚ versus SCLs or SVLs	151 (2)	0R = 4.56 (0.49, 42.25)	Favours SCLs/SVLs	0
Papillae/Follicles	2% pirenzepine gel versus control	323 (3)	OR = 3.21 (0.95, 10.88)	Favours control	74
Medication residue on eyelids or eye	2% pirenzepine gel versus control	323 (3)	OR = 0.77 (0.38, 1.59)	Favours pirenzepine	33
Abnormality of accommodation	2% pirenzepine gel versus control	323 (3)	OR = 16.92 (6.27, 45.64)	Favours control	0
Itching, eye	2% pirenzepine gel versus control	323 (3)	OR = 1.01 (0.54, 1.90)	No difference	0
Visual acuity decreased (subjectively)	2% pirenzepine gel versus control	323 (3)	OR = 3.89 (0.93, 16.27)	Favours control	33
Injection	2% pirenzepine gel versus control	323 (3)	OR = 0.92 (0.22, 3.73)	Favours pirenzepine	74
Fluorescein staining	2% pirenzepine gel versus control	323 (3)	OR = 0.57 (0.23, 1.44)	Favours pirenzepine	45
Burn/Sting, eye, on instillation	2% pirenzepine gel versus control	323 (3)	OR = 1.84 (0.76, 4.46)	Favours control	0
Eye/Vision, blurred	2% pirenzepine gel versus control	323 (3)	OR = 1.17 (0.52, 2.63)	Favours control	0
Erythema, eyelids	2% pirenzepine gel versus control	110 (2)	OR = 0.69 (0.01, 41.23)	Favours pirenzepine	76
Eyelid abnormality	2% pirenzepine gel versus control	110 (2)	OR = 1.73 (0.27, 11.12)	Favours control	0
Photophobia	2% pirenzepine gel versus control	110 (2)	OR = 1.57 (0.35, 6.96)	Favours control	0
Eye pain	2% pirenzepine gel versus control	110 (2)	OR = 2.07 (0.33, 12.98)	Favours control	0
Cough, increased	2% pirenzepine gel versus control	323 (3)	OR = 1.06 (0.59, 1.92)	No difference	0
Infection, respiratory	2% pirenzepine gel versus control	297 (2)	OR = 1.32 (0.69, 2.51)	Favours control	0
Rhinitis/Sinusitis	2% pirenzepine gel versus control	323 (3)	OR = 1.08 (0.42, 2.76)	No difference	28
Fever	2% pirenzepine gel versus control	297 (2)	OR = 1.07 (0.51, 2.24)	No difference	0
Abdominal pain	2% pirenzepine gel versus control	323 (3)	OR = 2.42 (0.88, 6.62)	Favours control	0
Headache	2% pirenzepine gel versus control	323 (3)	OR = 1.30 (0.66, 2.56)	Favours control	0
Flu syndrome	2% pirenzepine gel versus control	297 (2)	OR = 0.54 (0.26, 1.13)	Favours pirenzepine	0
Pharyngitis	2% pirenzepine gel versus control	323 (3)	OR = 1.07 (0.48, 2.37)	No difference	0
Rash/Allergic reaction	2% pirenzepine gel versus control	323 (3)	OR = 1.77 (0.51, 6.12)	Favours control	22
Cold, common	2% pirenzepine gel versus control	110 (2)	OR = 0.60 (0.25, 1.42)	Favours pirenzepine	0
Accidental injury	2% pirenzepine gel versus control	110 (2)	OR = 2.32 (0.74, 7.22)	Favours control	0

*CI* confidence interval, *OK* Orthokeratology, *OR* odds ratio, *RR* risk ratio, *SCLs* soft contact lenses, *SVLs* single vision lenses, *SVSCLs* single vision soft contact lense

Quality of the 44 index publications included in the meta-analysis was adequate. More than 50% of the RCTs were at low risk of bias for random sequence generation and allocation concealment and more than 80% of RCTs were at low risk of bias for selective outcome reporting. Nonetheless, only 20% of RCTs achieved appropriate blinding of participants and outcome assessors, while almost 70% were at high risk for incomplete outcome data. Three RCTs were assessed with Jadad scale and scored 4 or above, while one scored 2. More than 90% of included cohort studies were awarded with 8 or more stars in Newcastle-Ottawa Quality Assessment scale. Risk of bias assessments of index publications as presented in the eligible SRs/MAs are shown in Additional file 1: Tables S6, S7 and S8.

### Synthesis of results

The included SRs and MAs provided outcome data relating to the following comparisons: Undercorrected vs fully-corrected spectacles, bifocal spectacles vs single vision lens spectacles (SVLs), atropine vs placebo, pirenzepine gel vs placebo, rigid gas permeable contact lenses (RGPCLs) vs spectacles or soft contact lenses (SCLs), concentric ring bifocal SCLs vs single vision soft contact lenses (SVSCLs), peripheral add multifocal SCLs vs SCLs or SVLs, OK vs SCLs or SVLs, progressive addition lenses (PALs) vs SVLs. The outcomes assessed included change in refractive error and change in axial length from baseline to 1 year and from baseline to 2 years. These outcomes were identified a priori as being of interest for this overview [15]. Safety of myopia interventions was assessed by quantitative analysis of the number and type of reported adverse events.

### Effects of interventions

#### Undercorrected vs fully-corrected spectacles

Two RCTS encompassing 142 children investigated the effect of undercorrection in myopic progression. The overall pooled analysis revealed that the undercorrected group showed greater change in refractive error (RE) in 1 year (MD 0.15, 95% CI 0.00 to 0.29), and in 2 years from baseline (MD 0.20, 95% CI 0.01 to 0.39) and the evidence quality of this outcome was considered moderate (Tables 2, 3, 4 and 5 & Additional file 1: Appendices 4 and 6).

#### Bifocal spectacles vs single vision lens spectacles

Two RCTs (259 children) examined the effect of bifocal spectacles in myopia control and showed no change in RE in 1 year from baseline (MD -0.09, 95% CI -0.19 to 0.02; GRADE evidence: moderate; Table 2 & Additional file 1: Appendices 4 and 6). Three RCTs (351 children) reported no change in RE using bifocal spectacles in 2 years from baseline (MD -0.19, 95% CI -0.59 to 0.21; GRADE evidence: low; Table 3 & Additional file 1: Appendices 4 and 6). These 3 trials appeared to be inconsistent (*I*^*2*^ = 85%). Sensitivity analysis excluding *Parsinnen* et al. demonstrated no difference in the effect of bifocal spectacles.

#### 1% atropine vs placebo

Three RCTs (604 children) and three cohort studies (798 children) provided outcomes on the effect of 1% atropine eyedrops in refraction change in 1 year, (Table 2). Subgroup analysis of the three trials reported a change of − 0.78D, favouring atropine (95% CI, − 1.30 to − 0.25) with moderate quality of evidence (Additional file 1: Appendix 6). Due to high inconsistency (*I*^*2*^ = 97%), sensitivity analysis excluding *Yi* et al. revealed a change of − 0.54D, also favouring atropine (95% CI, − 0.76 to − 0.33), with moderate inconsistency among the two studies (*I*^*2*^ = 54%). Treatment effect reported by cohort studies showed an increase in refraction for the subgroup receiving placebo. Mean change in RE over 1 year was − 0.39D, favouring the use of atropine (95% CI, − 0.59 to − 0.19).

Two RCTs (540 children) and one cohort study (46 children) compared mean axial length (AL) change between 1% atropine eyedrops and placebo in 1 year (Table 4). Two trials revealed that atropine administration decreased AL change by − 0.35 mm (95% CI, − 0.38 to − 0.31). Treatment effect provided by the cohort study also favoured atropine, which showed AL change of − 0.61 mm (95% CI, − 0.88 to − 0.34). The overall treatment effect (586 children) showed that 1% atropine eyedrops can reduce AL change in 1 year (MD -0.36, 95% CI -0.41 to − 0.30), with moderate inconsistency among studies (*I*^*2*^ = 46%) and moderate evidence quality (Additional file 1: Appendix 6).

Two adverse events, including blurred near vision and allergic/hypersensitivity reactions or discomfort, were separately reported by two index publications. Two RCTs (540 children) showed that 1% atropine solution may induce blurred near vision (OR 9.47, 95% CI 1.17 to 76.78; Table 6 & Additional file 1: Appendices 5 and 6). One RCT and one cohort study (446 children) revealed an effect of 1% atropine for hypersensitivity reactions (OR 8.91, 95% CI 1.04 to 76.03), while no inconsistency exists between these two studies (*I*^*2*^ = 0%). One RCT (400 children) provided data on myopic progression and axial elongation for 2 years. Atropine appeared to reduce RE change (MD -0.92, 95% CI -1.08 to − 0.76) and favour AL change (MD -0.36, 95% CI -0.43 to − 0.29) compared to placebo (Tables 4 and 5 & Additional file 1: Appendices 4 and 6).

#### 0.025 To 0.05% atropine vs control

Three cohort studies (224 children) examined this comparison. An effect on refraction change in 1 year was reported (MD -0.51, 95% CI -0.60 to − 0.41), favouring atropine, while low inconsistency exists among these studies (*I*^*2*^ = 9%; Table 2 & Additional file 1: Appendices 4 and 6).

#### 0.01% atropine vs control

One cohort study (60 children) reported favourable effect of 0.01% atropine on RE change in 1 year (MD -0.50, 95% CI -0.76 to − 0.24, GRADE evidence quality: very low, Table 2 & Additional file 1: Appendices 4 and 6).

#### 2% Pirenzepine gel vs placebo

Two RCTs (264 children) examined the effect of pirenzepine in myopic progression. Findings showed that pirenzepine has a favourable effect on AL change, reducing it by − 0.10 mm in 1 year (95% CI, − 0.18 to − 0.01; GRADE evidence: moderate; Table 4 & Additional file 1: Appendix 6). Nonetheless, a number of reactions have been reported for this agent. Pirenzepine is more likely to induce abnormality of accommodation (OR 16.92, 95% CI 6.27 to 45.64) and subjectively reduce visual acuity (OR 3.89, 95% CI 0.93 to 16.27), while other adverse reactions had a smaller measure of effect (Additional file 1: Appendix 5). A full list of AE is provided in Table 6.

#### RGPCLs vs spectacles or SCLs

Two RCTs (420 children) failed to identify any effect of RGPCLs on myopic progression (Tables 2 and 4 & Additional file 1: Appendices 4 and 6). Although findings favour RGPCLs in reduction of refractive change, substantial inconsistency exists for both 1-year (*p* = 0.0008, *I*^*2*^ = 91%), and 2-year outcomes (*p* = 0.0005, *I*^*2*^ = 92%). Mean AL change did not differ between the two groups according to 1- and 2- year findings (Tables 3 and 5 & Additional file 1: Appendices 4 and 6).

#### Concentric ring bifocal SCLs vs SVSCLs

Three RCTs (264 children) showed an effect of concentric ring bifocal SCLs on myopia control, with low quality of evidence (Tables 2, 3, 4 and 5 & Additional file 1: Appendices 4 and 6). These trials reported a change of − 0.31D in 1 year, favouring concentric ring bifocal lenses (95% CI, − 0.60 to − 0.02). Due to high inconsistency (*p =* 0.0003, *I*^*2*^ = 88%), sensitivity analysis was performed. Exclusion of *Aller* et al. revealed a change of − 0.15D, favouring concentric ring bifocal lenses (95% CI, − 0.27 to − 0.03), with no existing inconsistency between studies (*p* = 0.35, *I*^*2*^ = 0%).The three trials (264 children) also compared mean AL change between concentric ring bifocal lenses and control in 1 year. Treatment with this type of lenses decreased AL change by − 0.12 mm (95% CI, − 0.19 to − 0.06). Two trials (261 children) reported contact lens-related discomfort or unwillingness to wear contact lenses (OR 0.95, 95% CI 0.49 to 1.81, Table 6 & Additional file 1: Appendices 5 and 6).

#### Peripheral add multifocal SCLs vs SCLs or SVLs

Two RCTs (105 children) and three cohort studies (189 children) examined this comparison. Subgroup analysis of two RCTs showed no change in refraction in 1 year (MD -0.13D, 95% CI -0.28 to 0.02), but revealed an effect in AL change in 1 year (MD -0.11, 95% CI -0.17 to − 0.05), favouring peripheral add multifocal lenses. Subgroup analysis of cohort studies revealed a treatment effect of multifocal lenses in refraction and AL change in 1 year, (MD -0.27D, 95% CI -0.38 to − 0.17) and (MD − 0.08 mm, 95% CI -0.16 to − 0.01), respectively. The overall treatment effect (294 children) showed that peripheral add multifocal lenses can slow refractive change in 1 year (MD -0.23D, 95% CI -0.31 to − 0.14), with no existing inconsistency among studies (*I*^*2*^ = 0%) and very low evidence quality (Table 2 & Additional file 1: Appendices 4 and 6). Two cohort studies (99 children) provided outcomes of the effect of peripheral add multifocal lenses in 2 years with very low evidence quality (Table 3 & Additional file 1: Appendices 4 and 6). Findings revealed that multifocal lenses can slow myopic progression, by reducing RE change (MD -0.50D, 95% CI -0.65 to − 0.36) and by restricting axial elongation (MD − 0.13 mm, 95% CI -0.20 to − 0.06).

#### OK vs SCLs or SVLs

Three RCTs (115 children) and 8 cohort studies (548 children) investigated the use of orthokeratology for myopia control. Subgroup analysis of two RCTs (113 children) showed a change of − 0.19 mm in axial elongation in 1 year, favouring OK (95% CI, − 0.25 to − 0.13). Similarly, subgroup analysis of six cohort studies (411 children) revealed favourable effect of OK in AL change in 1 year, which was reduced by − 0.18 mm (95% CI, − 0.22 to − 0.15). The overall treatment effect (524 children) with moderate evidence quality showed that OK can reduce AL change in 1 year by − 0.19 mm compared to control (95% CI, − 0.21 to − 0.16), with no inconsistency among studies (*I*^*2*^ = 0%; Table 4 & Additional file 1: Appendices 4 and 6). Three RCTs (108 children) investigated AL change in 2 years. Subgroup analysis of the clinical trials showed that mean AL change was − 0.27 mm, favouring OK (95% CI, − 0.36 to − 0.18). *Chan* et al. reported on each eye separately and due to unit of analysis issues, sensitivity analysis excluding *Chan* et al. revealed mean AL change of − 0.28 mm, favouring OK (95% CI, − 0.38 to − 0.19). Eight cohort studies (555 children) reported on the same outcome for 2 years of OK treatment. Subgroup analysis revealed that OK induced AL change of − 0.27 mm (95% CI, − 0.31 to − 0.22). Total effect of RCTs and cohort studies (663 children) revealed that OK restricts axial elongation (MD − 0.27 mm, 95% CI -0.31 to − 0.23), with no inconsistency among studies (*I*^*2*^ = 0%). Mild corneal erosion was reported by two cohort studies (151 children) as an adverse event (OR 4.56, 95% CI 0.49 to 42.25; Table 6).

#### PALs vs SVLs

Six trials (1151 children) provided moderate quality evidence on the effect of PALs in progression of myopia. The overall pooled analysis of two RCTs (206 children) showed that children treated with PALs achieved greater reduction in RE change in 1 year, (Table 2). Two RCTs (211 children) investigated the effect of PALs on AL change in 1 year, which was restricted by − 0.06 mm, favouring PALs (95% CI, − 0.12 to − 0.00; Table 4). Two-year results on refraction change were reported by four RCTs (940 children). PALs appeared to reduce RE change by − 0.26D (95% CI, − 0.39 to − 0.12), with moderate inconsistency among studies (*I*^*2*^ = 59%). Three RCTs (791 children) estimated AL change in 2 years. PALs induced a change of − 0.10 mm (95% CI -0.20 to 0.00) but with considerable inconsistency among studies (*I*^*2*^ = 78%). Sensitivity analysis excluding *Leung* et al. demonstrated no difference in this case.

## Discussion

This overview represents a comprehensive and thorough review of high level evidence from systematic reviews and meta-analyses on the efficacy and safety of optical and pharmaceutical modalities for restriction of myopic progression in children. Through this study, care was taken to identify and include all relevant methodologically robust primary studies and utilize them to perform an extensive meta-analysis, in order to fully depict current knowledge for retarding juvenile myopia. Owing to the reasonably limited number of published RCTs in this field so far, we incorporated high quality cohort studies in our analysis.

Existing high-level evidence suggests that atropine eyedrops appear to be more effective for myopia control compared to spectacles or CLs (Additional file 1: Table S9). Our findings are also in line with the consensus published by the World Society of Paediatric Ophthalmology and Strabismus (WSPOS), which reported that atropine is the most beneficial intervention for myopia progression control [40]. In addition, modern orthokeratology also demonstrates efficacy in retarding myopia development compared to other types of lenses [10, 41], though its use is considerably limited by the associated high risk for microbial keratitis [42, 43]. Multifocal CLs designed with novel technology appear as an emerging treatment which has also proved to be effective, and has a low reported risk for infectious keratitis [11]. Finally, there is increasing evidence that outdoor exposure in children has a protective effect on myopia development and should be readily encouraged [12].

Despite the apparent beneficial effect of atropine, it has not been widely adopted for myopia treatment [28, 29]. Atropine eyedrops (1%) have been approved by U.S. Food and Drug Administration (FDA) for amblyopia treatment, but not for myopia control, and a diluted preparation (0.01%) does not exist in the market in most countries. Α drawback of atropine is the fact that myopic patients still need spectacles or contact lenses for good distant vision, while the combination of atropine with multifocal or bifocal spectacles has not shown any advantage [44]. There exists a relatively small, but not scarce, subgroup of myopic individuals who do not respond to this treatment. Notably lacking is an evidence-based and widely accepted management plan that would define indications for treatment, timing of initiation and discontinuation, taking into account age, severity of myopia, rate of myopia progression, family history of myopia, race etc. [28]. Wu et al. proposed a treatment strategy for myopia control with the use of 0.01% atropine solution. Authors advocated initial treatment with atropine for 2 years and in case of rapid progress, combination of atropine with time outdoors, stepwise increase in concentration or implementation of alternative therapy, such as orthokeratology. Decision on continuation of treatment after 2 years relied on the myopia progression rate. However, uncertainty still remains regarding poor responders, as well as treatment duration and whether a wash-out period is deemed necessary [45].

When it comes to optimal atropine dose choice, findings from our meta-analysis are concordant with recent evidence from a network meta-analysis in 2016 [34] and another meta-analysis in 2017 [9] which showed no dose dependence and no difference in the efficacy of atropine across different doses in the range of 0.01–1%. Nonetheless, latest findings from Phase 1 of the LAMP study unveiled a concentration-dependent pattern of decelerating myopic progression among low dosages (0.01–0.05%) of atropine. These 1-year findings demonstrated that 0.01% atropine was effective in reducing refractive change, but not in restricting axial elongation [46]. Concordant results after 1 year of follow-up had been previously reported by ATOM 2 study [47]. LAMP proposed the use of 0.05% atropine as an optimal dose for obtaining clinically important outcomes, with a minimum risk for adverse reactions including photophobia, reduction in accommodative amplitude and pupillary dilation [46, 48]. Notwithstanding, five-year results from ATOM2 study supported binocular daily application of 0.01% atropine as the safest and most effective concentration for restricting myopia, as it appears that a plateau effect occurs following prolonged use of atropine with regard to clinically meaningful results [47]. Furthermore, higher doses of atropine have been associated with increased risk for adverse events, such as photophobia, poor near visual acuity, allergy and rebound effect [9, 47]. An inverse dose-related rebound effect upon treatment discontinuation has also been described [28]. Pirenzepine, which acts only to M1 anti-muscarinic receptors that are less concentrated in ciliary body and iris, is believed to have a lower impact on dilatation of the pupil or accommodation compared to atropine. Despite the encouraging findings shown by two RCTs, research on this agent has been abandoned, due to related costs and regulatory purposes [49, 50]. Further research in this area is warranted to investigate long-term efficacy of lower atropine concentrations, long-term adverse reactions, as well as the rebound phenomenon [48].

Modern orthokeratology has been described as a major effective alternative to atropine for myopia treatment. Orthokeratology lenses are worn overnight and provide the advantage of clear vision during the day without the need for optical correction. Findings from a recent RCT showed that stopping OK use after 2 years of treatment results in greater axial growth compared to individuals who continued treatment, but similar to those who wore spectacles during this 2-year period. Interestingly, axial elongation was retarded after resuming the lenses for a 6-month period. However, more evidence on the effect of OK is needed [51], especially with regards to its safety whereby major concerns have been raised [10, 32]. A recent systematic review reported on the infectious keratitis clinical profile following OK lens use. The study included 173 eyes of 166 patients with this complication and suggested that in spite of early treatment, most infections caused formation of corneal scars and nearly 10% of the cases required surgical treatment [43]. Robust evidence on the overall incidence of keratitis was not available. Another systematic review on the safety of OK reported corneal staining as the most prominent side effect, along with lens binding and reduced tear film stability in long-term use. Orthokeratology side effects have resulted in this treatment presenting higher dropout rates compared to other myopia interventions. Patient training on proper fitting of the lenses and advice on timely attendance in case signs of ocular infection appear, is crucial [33, 39, 42, 43].

The use of modern multifocal soft CLs designed with novel technology has also been recently highlighted for myopia management. Initially, a number of clinical studies were conducted on bifocal lenses for myopia control. These lenses incorporate two parts for distance and near vision, which are clearly demarcated and, therefore, produce a prismatic effect [52]. In accord, modern types of concentric ring bifocal soft CLs consist of a center-distance zone enclosed by multiple rings of power with near addition, while peripheral add multifocal soft CLs are made of center-distance zone surrounded by progressively increasing power which gradually becomes positive in the periphery. The design of multifocal lenses is based on the imposition of myopic defocus at all distances, which aims to employ emmetropisation process so as to retard progression of myopia [53, 54]. Multifocal CLs demonstrate lower risk of ocular infections compared to overnight lenses. Multifocal spectacles have also been reported to produce similar outcomes [11]. Findings of the RCT investigating multiple segment (MS) spectacle lenses (NCT02206217) will provide more clinical data on this intervention. Future designs of multifocal lenses should aim to provide higher retinal image quality [11].

Increased outdoor exposure is yet another myopia-controlling intervention for which the mechanism of action has not been clarified. Index publications assessing outdoor exposure are not statistically analysed in this overview, due to serious limitations of the studies assessing this intervention: a) outcome measures and study design vary largely between these studies and additionally outcomes are distinct from ours, b) a number of them have broad age range of participants involving adults, c) observational studies present several types of biases such as recall bias and loss to follow-up, finally d) synthesizing evidence from RCTs and observational studies, mainly cross-sectional ones, would probably provide imprecise estimates. Lastly, current evidence on the effect of outdoor exposure reflects controversy. A systematic review and meta-analysis analysing up-to-date evidence showed that outdoor exposure appears to provide protection from myopia onset in nonmyopes, but does not result in restriction of myopia progression in already myopic individuals [12]. In contrast, a recent RCT reports a beneficial effect of outdoor exposure in both nonmyopic and myopic individuals [55]. Additional evidence on this area is expected from clinical trials underway (NCT02980445, NCT03552016).

Optical undercorrection has been another debatable issue, as studies have produced contradictory results over the years. Our meta-analysis showed that full correction reduces progression of myopia compared to undercorrection over a 2-year period of treatment [56, 57]. A retrospective cohort study by Vasudevan et al. also supports this finding [58]. Nonetheless, a recent cohort study on 121 Chinese children proposed that abstinence from correction is effective in slowing myopic progression and axial elongation compared to full correction [59], which is in line with former findings from animal studies [60, 61]. Undercorrection on animal models imposes myopic defocus which was considered to slow myopic progression. This intervention proved effective in animals, possibly because it was implemented at a very early stage of development, in contrast to the majority of human studies [58].

To our knowledge, this is the first overview of systematic reviews and meta-analyses on interventions for myopia control. Through this study, we identified and synthesized all available high level evidence, estimated the actual overlap of index publications that composed eligible reviews, and reported on efficacy and safety of myopia interventions. Certain limitations stand out in this overview. A number of treatments, such as atropine and OK, were represented by a larger number of reviews compared to other therapies, including bifocal or multifocal lenses. In one large SR [5], dual co-authorship was identified, as two of the authors were principal investigators in two included trials and both of them were involved in quality assessment of the included index publications. A protocol was not available for the majority of eligible reviews, and one protocol amendment was reported [5]. A large proportion of the eligible index publications contained in the systematic reviews were at high risk of bias for selective outcome reporting. Publication bias was suspected in eleven reviews, due to language restrictions and exclusion of unpublished material or conference abstracts. Included index publications were largely unable to achieve appropriate blinding and allocation concealment, mainly due to the nature of the investigated interventions (eyedrops, spectacles, contact lenses). Follow-up periods varied significantly among the trials, and losses to follow-up were also noted, mainly depending on the type of treatment and related adverse events. The majority of index publications were conducted in Asian ethnicities, which could compromise the external validity of their findings. Due to small sample sizes analysed, treatment effects are likely to be overestimated. Index publications either reported on one affected eye, or each eye separately, or provided the measure of effect as the average of both eyes [62]. Finally, only 9 index publications reported on adverse events.

## Conclusions

Our data suggest that atropine followed by orthokeratology and novel multifocal soft contact lenses demonstrate efficacy in controlling myopic progression. Future research should be geared towards effective interventions and their potential combinations. More evidence on low-dose atropine is needed and several parameters remain to be defined, such as the appropriate onset and duration of treatment, as well as the period needed for tapering off the medication without causing a rebound effect. ATOM3 study (NCT03140358) is underway and is expected to provide some answers to outstanding issues. It remains unclear if atropine or orthokeratology could lead to a permanent long-term effect on myopia control. Possible rebound effect upon treatment cessation should also be assessed for OK and multifocal lenses. In addition, more research in non-Asian ethnicities is needed. Methodologically rigorous trials with long-term follow-up and large sample sizes constitute the optimal study design for further investigating myopia interventions. Finally, systematic collection of evidence on safety issues is essential, as these treatments gradually enter routine practice all over the world.

## Additional file

Additional file 1: “Efficacy and safety of interventions to control myopia progression in children: An overview of systematic reviews and meta-analyses.” - includes Appendices and Tables pertaining to the search strategy, forest plots, citation matrix, methodological quality assessment and a summary of the findings of each included study. (DOCX 662 kb)

## Abbreviations

AE: Adverse events
AL: Axial length
CCA: Corrected Covered Area
CLs: Contact lenses
CRD: Centre for Reviews and Dissemination
DARE: Database of Abstracts of Reviews of Effects
FDA: Food and Drug Administration
HTA: Health Technology Assessment
MAs: Meta-analyses
MeSH: Medical subject headings
OK: Orthokeratology
PALs: Progressive addition lenses
PRIO: Preferred Reporting Items for Overviews
PROSPERO: International prospective register of systematic reviews
RCTs: Randomized Controlled Trials
RGPSCLs: Rigid gas permeable soft contact lenses
ROBIS: Risk of Bias in Systematic Reviews
SCLs: Soft contact lenses
SRs: Systematic reviews
SVLs: Single vision lens spectacles
SVSCLs: Single vision soft contact lenses
