# Supplementary material for: Efficacy and safety of interventions to control myopia progression in children: an overview of systematic reviews and meta-analyses
Source: BMC Ophthalmol. 2019 May 9;19:106. doi: 10.1186/s12886-019-1112-3 (PMC6506938; doi:10.1186/s12886-019-1112-3)
Supplement: Supplementary file 1 — “Efficacy and safety of interventions to control myopia progression in children: An overview of systematic reviews and meta-analyses.” - includes Appendices and Tables pertaining to the search strategy, forest plots, citation matrix, methodological quality assessment and a summary of the findings of each included study. (DOCX 662 kb) [file 12886_2019_1112_MOESM1_ESM.docx]

**Additional File 1**

**Efficacy and safety of interventions to control myopia progression in children: An overview of systematic reviews and meta-analyses.**

Efthymia Prousali, Anna-Bettina Haidich, Andreas Fontalis, Nikolaos Ziakas, Periklis Brazitikos, Asimina Mataftsi

February 5, 2019

**Appendix 1.** Preferred Reporting Items for Overviews of systematic reviews with harms 2017 Checklist

**Appendix 2.** Search strategy

**Appendix 3.** Included primary studies contained within systematic reviews and meta-analyzed.

**Appendix 4.** Forest plots of comparisons.

**Appendix 5.** Forest plots of adverse events.

**Appendix 6.** GRADE assessment of Overall Strength of Evidence.

**Table S1.** Handling of heterogeneity & meta-biases.

**Table S2.** Unit of analysis for RCTs included in the meta-analysis.

**Table S3.** Excluded studies after full text review.

**Table S4.** Citation matrix.

**Table S5.** Methodological quality of included systematic reviews and meta-analyses based on ROBIS tool.

**Table S6.** Methodological quality of included RCTs assessed using the Cochrane Collaboration Risk of Bias tool.

**Table S7.** Methodological quality of included RCTs assessed using Jadad Scale.

**Table S8.** Methodological quality of included cohort studies assessed using Newcastle-Ottawa Quality Assessment Scale.

**Table S9.** Summary of qualitative and quantitative findings of each study.

**Appendix 1. P**referred **R**eporting **I**tems for **O**verviews of systematic reviews with harms 2017 Checklist

| **Section/Topic** | **(Sub-) item #** | **Checklist item** | | | | | **Reported**  **on page #** |
| --- | --- | --- | --- | --- | --- | --- | --- |
| **TITLE** | | | | | | |  |
| 1. Title | 1a | Specify the study design with terms such as “overview of (systematic) reviews,” “umbrella review,” “(systematic) review of systematic reviews,” or “(systematic) meta-review” in the title of the OoSRs. | | | | | 1 |
|  | 1b | Mention “safety” or harms related terms, or the adverse event(s) of interest in the title of the OoSRs. | | | | | 1 |
| **ABSTRACT** | | | | | | |  |
| 2. Structured-like summary | 2a | Provide a structured-like abstract, as applicable: background, objective, data sources, selection criteria, data extraction, review appraisal, data synthesis methods, results, limitations, conclusions. | | | | | 2 |
|  | 2b | Report the main findings of analysis of harms undertaken in the OoSRs or/and in the included SRs. | | | | | 2 |
| **INTRODUCTION** | | | | | | |  |
| 3. Rationale | 3a | Specify the rationale and the scope (wide or narrow agendas) for the overview in the context of an existing body of knowledge on the topic. | | | | | 3 |
|  | 3b | Provide a balanced presentation of potential benefits and harms of the intervention(s). | | | | | 3 |
|  | 3c**^a^** | Define which events are considered harms according to previous literature and provide a clear rationale for the specific harms included in the OoSRs. | | | | | 3 |
| 4. Objectives  (PICOS) | 4 | Provide an explicit statement of research question(s) that specifies PICOS: | | | | | 5 |
|  |  | \|  \| \| --- \|  - Participants | \|  \| \| --- \|  - Interventions | \|  \| \| --- \|  - Comparators | \|  \| \| --- \|  - Outcomes | \|  \| \| --- \|  - Study design |  |
| **METHODS** | | | | | | |  |
| 5. Protocol and registration | 5a | Indicate clearly if a protocol exists or not. | | | | | 4 |
|  | 5b | If registered, provide the name of the registry (such as a valid Web address, PROSPERO). | | | | | 4 |
| 6. Eligibility criteria  & outcomes of interest | 6a | Specify inclusion and exclusion criteria for study design, participants, interventions and comparators in detail. | | | | | 5-6 |
|  | 6b | List (and define whenever it is necessary) the outcomes for which data were recorded, ideally include prioritization of main and additional outcomes. | | | | | 5 |
|  | 6c | Include adverse events as (primary or secondary) outcome of interest. Define them and grade their severity (such as mild, moderate, severe, fatal; severity could also be described in the appendix), if appropriate. | | | | | 5 |
|  | 6d**^b^** | Specify report characteristics (such as language restrictions, publication status, and years considered) used as criteria for eligibility for the OoSRs (see also item 7). | | | | | 4 |
| 7. Information sources | 7a | Search at least two electronic bases. | | | | | 4 |
|  | 7b | Search supplementary sources (e.g. hand-searching, reference lists, related reviews and guidelines, protocol registries, conference abstracts, and other gray literature). | | | | | 4 |
|  | 7c | Report the date last searched and/or dates of coverage for each database. | | | | | 4 |
| 8. Search strategy**^c^** | 8a | Specify full electronic search strategy (algorithm) for at least one database including any limits used (e.g. language and date restrictions-see also subitems 6d and 7c) such that it could be repeated. | | | | | Appendix 2 |
|  | 8b | Present any additional search process (e.g. algorithm or filter for adverse events, searches in pertinent websites) specifically to identify adverse events that have been investigated. | | | | | 4 |
| 9. Data management & selection process | 9a**^d^** | Describe the software that was used to manage records and data throughout the OoSRs. | | | | | 6 |
|  | 9b | Define what is a SR and provide the process for selecting SRs and its relevant details (screening the title and abstract or full text by at least two reviewers, selection by multiple independent investigators and resolving disagreements by consensus). | | | | | 6 |
|  | 9c | Report any attempt to handle overlapping (include one review among multiple potential candidates by choosing for example the most updated SR, the most methodologically rigorous SR or the SR with larger number of primary studies). | | | | | 7 |
| 10. Additional search for primary studies | 10 | Report additional search to identify eligible primary studies (e.g. searching in more databases or update the search) and its relevant details. | | | | | 4 |
| 11. Data collection process | 11a | Describe the method of data extraction from included SRs (e.g. data collection form, extraction in duplicate and independently, resolving disagreements by consensus). | | | | | 6 |
|  | 11b | Report any processes for obtaining, confirming or updating data from investigators (e.g. contact with authors of included reviews, obtain data from primary studies of included reviews). | | | | |  |
| 12. Data items | 12 | List (and define whenever is necessary) the specific variables for which data were recorded (e.g. PICOS items, number of included studies and participants, dose, length of follow up, results, funding sources) and any data assumptions and simplifications made. | | | | | Protocol |
| 13. Assessment of methodological quality & quality of evidence | 13a | State the evaluation of reporting or/and methodological quality (eg. using PRISMA or PRISMA-harms, AMSTAR or R-AMSTAR) of the included reviews. | | | | | 7 |
|  | 13b**^e^** | State the evaluation of quality for individual studies that were included in the SRs (inform whether tools such as Jadad or RoB of Cochrane were used by the included reviews) and for the additional primary studies. | | | | | 5 |
|  | 13c | State the evaluation of quality of evidence (e.g. using GRADE approach). | | | | | 7 |
|  | 13d | Describe the methods (e.g. piloted forms, independently, in duplicate) used for the quality assessment. | | | | | 6-8 |
| 14. Meta-bias(es) | 14 | Specify any planned assessment of meta-bias(es) (such as publication bias or selective reporting across studies, ROBIS tool). | | | | | 7 |
| 15. Data synthesis | 15a | Specify clearly the method (narrative, meta-analysis or network meta-analysis) of handling or synthesizing data and their details (e.g. state the principal summary measures that were extracted or calculated, how heterogeneity was assessed, what statistical approaches were used if a quantitative synthesis has been conducted). | | | | | 8 |
|  | 15b | Describe the software that was used to analyze the data if a quantitative synthesis has been conducted. | | | | | 8 |
|  | 15c | Report if zero events are included in the studies and how they were handled in statistical analyses, if relevant. | | | | |  |
|  | 15d | Describe methods of any pre-specified additional analyses (such as sensitivity or subgroup analyses, meta-regression). | | | | | 8 |
| **RESULTS** | | | | | | |  |
| 16. Review & primary study selection | 16a | Provide the details of review selection (e.g. numbers of reviews screened, retrieved, and included and excluded in the overview) and the number of the additional eligible primary studies that were included, ideally with a flow diagram of the overview process. | | | | | 8-9 |
|  | 16b | Present a flow diagram that gives separately the number of studies focused on harms outcomes. | | | | | Figure 1 |
|  | 16c**^c^** | List the studies (full citation) that were excluded after reading the full text and provide reasons. | | | | | Table S3 |
| 17. Review & primary study characteristics | 17a**^c^** | Describe characteristics of each included SR in tables (such as title or author, search date, PICOS, design and number of studies included, number and age range of participants, dose/frequency, follow up period [treatment duration], review limitations, results or conclusion) and of each additional primary study. | | | | | Table 1 |
|  | 17b | For each included SR report language and publication status restrictions that have been used. | | | | | 8-9 |
| 18. Overlapping | 18 | Present or/and discuss about overlapping of studies within SRs (at least one of the following): | | | | | 13-14 |
|  |  | - Present measures of overlap (such as CCA). | | | | | 13-14 |
|  |  | - Provide citation matrix.**^c^** | | | | | Table S4 |
|  |  | - Give the number of index publications or/and discuss about overlapping.**^f^** | | | | | 13-14 |
| 19. Present assessment of methodological quality & quality of evidence | 19 | Present results in text or/and tables**^c^** of any quality assessment (see also subitems 13a-c): | | | | | 13-14, Tables 2-6 |
|  |  | - Reporting or/and methodological quality of the included SRs. | | | | | 13-14,  Table S5 |
|  |  | - Inform for the quality of the individual studies that were included in the SRs (report results for sequence generation, allocation concealment, blinding, withdrawals, bias etc.) and for the additional included primary studies. | | | | | 13-14,  Tables  S6-8 |
|  |  | - Quality of evidence. | | | | | Appendix 6 |
| 20. Present meta-bias(es) | 20 | Present results of any assessment of meta-bias(es) (such as publication bias or selective reporting across studies, ROBIS assessment). | | | | | Table S1 |
| 21. Synthesis of results | 21a | Summarize and present the main findings of the overview for benefits and harms. If a quantitative synthesis has been conducted, present each summary measure with a confidence interval, prediction interval or a credible interval and measures of heterogeneity or inconsistency. | | | | | 14-24 |
|  | 21b | Give results of any additional analyses (such as sensitivity, subgroup analyses, or meta-regression). | | | | | 14-24 |
|  | 21c | Report results for adverse events separately for each intervention. | | | | | 14-24 |
| **DISCUSSION** | | | | | | |  |
| 22. Summary of evidence | 22 | Provide a concise summary of the main findings with the strength and shortcomings of evidence for each main outcome. | | | | | 25-30 |
| 23. Limitations | 23a | Discuss limitations of either the overview or included studies (or both) (e.g. different eligibility criteria, limitations of searching reviews, language restrictions, publication and selection bias). | | | | | 25-30 |
|  | 23b | Report possible limitations of the included reviews related to harms (issues of missing data and information, definitions of harms, rare adverse effects). | | | | | 25-30 |
| 24. Conclusions | 24a | Provide a general interpretation of the results in coherence with the review findings and present implications for practice; consider the harms equally as carefully as the benefits and in the context of other evidence. | | | | | 25-30 |
|  | 24b | Present implications for future research. | | | | | 30 |
| **AUTHORSHIP** | | | | | | |  |
| 25. Contributions of authors | 25 | Provide contributions of authors. | | | | | 32 |
| 26. Dual (co-)authorship | 26 | Report about dual (co-)authorship in the limitation or declarations of interest section. | | | | |  |
| **FUNDING** | | | | | | |  |
| 27. Funding or other support | 27a | Indicate sources of financial and other support for the OoSRs (direct funding) or for the authors (indirect funding), or report no funding. | | | | | 31-32 |
|  | 27b | Provide name for the overview funder and/or sponsor, or for the authors’ supporters. | | | | | 31-32 |
|  | 27c | Describe roles of funder(s), sponsor(s), and/or institution(s), if any, in conducted the OoSRs. | | | | | 31-32 |

***Abbreviations:*** *CCA, corrected covered area; N/A, not applicable; OoSRs, Overview of Systematic Reviews; PICOS, participants, interventions, comparisons, outcomes, and study design; SRs, Systematic Reviews*

^a^Applicable mainly for OoSRs that focus on adverse events. The description could be placed in methods section.

^b^Language restrictions, publication status, and years could also be reported in information sources topic—see item 7.

^c^It could also be placed in an appendix as a supplementary material.

^d^The software used for the management of the records and data could be placed in the data collection process—see item 11.

^e^The way of evaluation (e.g. instruments) can be reported in item 19.

^f^Index publication is the first occurrence of a primary publication in the included reviews. Discussion for overlapping might be placed in the discussion section.

*Modified and extended for Overviews of Systematic Reviews (OoSRs) from:*  Moher D, Liberati A, Tetzlaff J, Altman DG, The PRISMA Group (2009). Preferred Reporting Items for Systematic Reviews and Meta-Analyses: The PRISMA Statement. PLoS Med 6(7): e1000097. <https://doi.org/10.1371/journal.pmed.1000097>

**Appendix 2. Search strategy**

**MEDLINE Search algorithm**

| 1. exp MYOPIA/ |
| --- |
| 1. (myop*).ti,ab |
| 1. (shortADJ3sight*).ti,ab |
| 1. (1 OR 2 OR 3) |
| 1. exp EYEGLASSES/ |
| 1. (spectacles OR glasses).ti,ab |
| 1. exp “CONTACT LENSES”/ |
| 1. (contactADJ2lens*).ti,ab |
| 1. exp “MUSCARINIC ANTAGONISTS”/ |
| 1. (muscarinicADJ2antagonist*).ti,ab |
| 1. (antiADJ1muscarinic).ti,ab |
| 1. exp “CHOLINERGIC ANTAGONISTS”/ |
| 1. (cholinergicADJ2antagonist*).ti,ab |
| 1. (antiADJ1cholinergic).ti,ab |
| 1. exp ATROPINE/ |
| 1. (atropine*).ti,ab |
| 1. exp CYCLOPENTOLATE/ |
| 1. (cyclopentolate*).ti,ab |
| 1. exp PIRENZEPINE/ |
| 1. (pirenzepine*).ti,ab |
| 1. exp TROPICAMIDE/ |
| 1. (tropicamide*).ti,ab |
| 1. exp TIMOLOL/ |
| 1. (timolol*).ti,ab |
| 1. exp PHENYLEPHRINE/ |
| 1. (phenylephrine*).ti,ab |
| 1. (5 OR 6 OR 7 OR 8 OR 9 OR 10 OR 11 OR 12 OR 13 OR 14 OR 15 OR 16 OR 17 OR 18 OR 19 OR 20 OR 21 OR 22 OR 23 OR 24 OR 25 OR 26) |
| 1. (4 AND 27) |
| 1. exp INFANT/ |
| 1. (infant*).ti,ab |
| 1. (infancy).ti,ab |
| 1. (newborn*).ti,ab |
| 1. (baby*).ti,ab |
| 1. (babies).ti,ab |
| 1. (neonat*).ti,ab |
| 1. (preterm*).ti,ab |
| 1. (prematur*).ti,ab |
| 1. (postmatur*).ti,ab |
| 1. exp CHILD/ |
| 1. (child*).ti,ab |
| 1. (schoolchild*).ti,ab |
| 1. (school age*).ti,ab |
| 1. (preschool*).ti,ab |
| 1. (kid*).ti,ab |
| 1. (toddler*).ti,ab |
| 1. exp ADOLESCENT/ |
| 1. (adoles*).ti,ab |
| 1. (teen*).ti,ab |
| 1. (boy*).ti,ab |
| 1. (girl*).ti,ab |
| 1. exp MINORS/ |
| 1. (minors*).ti,ab |
| 1. exp PUBERTY/ |
| 1. (pubert*).ti,ab |
| 1. (pubescen*).ti,ab |
| 1. (prepubscen*).ti,ab |
| 1. exp PEDIATRICS/ |
| 1. (pediatric*).ti,ab |
| 1. (paediatric*).ti,ab |
| 1. (peadiatric*).ti,ab |
| 1. exp SCHOOLS/ |
| 1. (nursery school*).ti,ab |
| 1. (kindergar*).ti,ab |
| 1. (primary school*).ti,ab |
| 1. (secondary school*).ti,ab |
| 1. (elementary school*).ti,ab |
| 1. (high school*).ti,ab |
| 1. (highschool*).ti,ab |
| 1. (29 OR 30 OR 31 OR 32 OR 33 OR 34 OR 35 OR 36 OR 37 OR 38 OR 39 OR 40 OR 41 OR 42 OR 43 OR 44 OR 45 OR 46 OR 47 OR 48 OR 49 OR 50 OR 51 OR 52 OR 53 OR 54 OR 55 OR 56 OR 57 OR 58 OR 59 OR 60 OR 61 OR 62 OR 63 OR 64 OR 65 OR 66 OR 67 OR 68) |
| 1. (28 AND 69) |

**Appendix 3.**

**Included primary studies contained within systematic reviews and meta-analysed.**

1. Adler, D. & Millodot, M. The possible effect of undercorrection on myopic progression in children. *Clin. Exp. Optom.* 89, 315–321 (2006).

2. Aller, T. A., Liu, M. & Wildsoet, C. F. Myopia Control with Bifocal Contact Lenses: A Randomized Clinical Trial. *Optom. Vis. Sci.* 93, 344–52 (2016).

3. Anstice, N. S. & Phillips, J. R. Effect of dual-focus soft contact lens wear on axial myopia progression in children. *Ophthalmology* 118, 1152–1161 (2011).

4. Bartlett, J. D. *et al.* A tolerability study of pirenzepine ophthalmic gel in myopic children. *J. Ocul. Pharmacol. Ther.* 19, 271–279 (2003).

5. Berntsen, D. A., Sinnott, L. T., Mutti, D. O. & Zadnik, K. A randomized trial using progressive addition lenses to evaluate theories of myopia progression in children with a high lag of accommodation. *Invest. Ophthalmol. Vis. Sci.* 53, 640–649 (2012).

6. Brodstein, R. S., Brodstein, D. E., Olson, R. J., Hunt, S. C. & Williams, R. R. The treatment of myopia with atropine and bifocals. A long-term prospective study. *Ophthalmology* 91, 1373–1379 (1984).

7. Chan, K. Y., Cheung, S. W. & Cho, P. Orthokeratology for slowing myopic progression in a pair of identical twins. *Cont. Lens Anterior Eye* 37, 116–119 (2014).

8. Charm, J. & Cho, P. High myopia-partial reduction ortho-k: a 2-year randomized study. *Optom. Vis. Sci.* 90, 530–539 (2013).

9. Chen, C., Cheung, S. W. & Cho, P. Myopia control using toric orthokeratology (TO-SEE study). *Invest. Ophthalmol. Vis. Sci.* 54, 6510–6517 (2013).

10. Cheng, D., Schmid, K. L., Woo, G. C. & Drobe, B. Randomized trial of effect of bifocal and prismatic bifocal spectacles on myopic progression: two-year results. *Arch. Ophthalmol. (Chicago, Ill. 1960)* 128, 12–9 (2010).

11. Cheng, X., Xu, J., Chehab, K., Exford, J. & Brennan, N. Soft Contact Lenses with Positive Spherical Aberration for Myopia Control. *Optom. Vis. Sci.* 93, 353–366 (2016).

12. Cho, P., Cheung, S. W. & Edwards, M. The longitudinal orthokeratology research in children (LORIC) in Hong Kong: a pilot study on refractive changes and myopic control. *Curr. Eye Res.* 30, 71–80 (2005).

13. Cho, P. & Cheung, S.-W. Retardation of myopia in Orthokeratology (ROMIO) study: a 2-year randomized clinical trial. *Investig. Ophthalmol. Vis. Sci.* 53, 7077–7085 (2012).

14. Chua, W.-H. *et al.* Atropine for the treatment of childhood myopia. *Ophthalmology* 113, 2285–2291 (2006).

15. Chung, K., Mohidin, N. & O’Leary, D. J. Undercorrection of myopia enhances rather than inhibits myopia progression. *Vision Res.* 42, 2555–2559 (2002).

16. Clark, T. Y. & Clark, R. A. Atropine 0.01% Eyedrops Significantly Reduce the Progression of Childhood Myopia. *J. Ocul. Pharmacol. Ther.* 31, 541–545 (2015).

17. Edwards, M. H. *et al.* The Hong Kong progressive lens myopia control study: Study design and main findings. *Invest. Ophthalmol. Vis. Sci.* 43, 2852–2858 (2002).

18. Fan, D. S. P. *et al.* Topical atropine in retarding myopic progression and axial length growth in children with moderate to severe myopia: a pilot study. *Jpn. J. Ophthalmol.* 51, 27–33 (2007).

19. Fang, P.-C. *et al.* Prevention of myopia onset with 0.025% atropine in premyopic children. *J. Ocul. Pharmacol. Ther.* 26, 341–345 (2010).

20. Fujikado, T. *et al.* Effect of low-addition soft contact lenses with decentered optical design on myopia progression in children: a pilot study. *Clin. Ophthalmol.* 8, 1947–1956 (2014).

21. Gwiazda, J. *et al.* A randomized clinical trial of progressive addition lenses versus single vision lenses on the progression of myopia in children. *Invest. Ophthalmol. Vis. Sci.* 44, 1492–1500 (2003).

22. Hiraoka, T., Kakita, T., Okamoto, F., Takahashi, H. & Oshika, T. Long-term effect of overnight orthokeratology on axial length elongation in childhood myopia: a 5-year follow-up study. *Invest. Ophthalmol. Vis. Sci.* 53, 3913–3919 (2012).

23. Jensen, H. Myopia progression in young school children. A prospective study of myopia progression and the effect of a trial with bifocal lenses and beta blocker eye drops. *Acta Ophthalmol. Suppl. (Oxf. ).* 1–79 (1991).

24. Kakita, T., Hiraoka, T. & Oshika, T. Influence of overnight orthokeratology on axial elongation in childhood myopia. *Invest. Ophthalmol. Vis. Sci.* 52, 2170–2174 (2011).

25. Katz, J. *et al.* A randomized trial of rigid gas permeable contact lenses to reduce progression of children’s myopia. *Am. J. Ophthalmol.* 136, 82–90 (2003).

26. Kennedy, R. H. *et al.* Reducing the progression of myopia with atropine: a long term cohort study of Olmsted County students. *Binocul. Vis. Strabismus Q.* 15, 281–304 (2000).

27. Lam, C. S. Y., Tang, W. C., Tse, D. Y.-Y., Tang, Y. Y. & To, C. H. Defocus Incorporated Soft Contact (DISC) lens slows myopia progression in Hong Kong Chinese schoolchildren: a 2-year randomised clinical trial. *Br. J. Ophthalmol.* 98, 40–45 (2014).

28. Lee, J.-J. *et al.* Prevention of myopia progression with 0.05% atropine solution. *J. Ocul. Pharmacol. Ther.* 22, 41–46 (2006).

29. Leung, J. T., Brown, B., J.T.M., L. & B., B. Progression of myopia in Hong Kong Chinese schoolchildren is slowed by wearing progressive lenses. *Optom. Vis. Sci.* 76, 346–354 (1999).

30. Pärssinen, O. *et al.* Effect of spectacle use and accommodation on myopic progression: Final results of a three-year randomised clinical trial among schoolchildren. *Br. J. Ophthalmol.* 73, 547–551 (1989).

31. Pauné, J. *et al.* Myopia Control with a Novel Peripheral Gradient Soft Lens and Orthokeratology: A 2-Year Clinical Trial. *Biomed Res. Int.* 2015, 507572 (2015).

32. Sankaridurg, P. *et al.* Spectacle lenses designed to reduce progression of myopia: 12-month results. *Optom. Vis. Sci.* 87, 631–641 (2010).

33. Sankaridurg, P. *et al.* Decrease in rate of myopia progression with a contact lens designed to reduce relative peripheral hyperopia: one-year results. *Invest. Ophthalmol. Vis. Sci.* 52, 9362–9367 (2011).

34. Shih, Y. F. *et al.* An intervention trial on efficacy of atropine and multi-focal glasses in controlling myopic progression. *Acta Ophthalmol. Scand.* 79, 233–236 (2001).

35. Siatkowski, R. M. *et al.* Two-year multicenter, randomized, double-masked, placebo-controlled, parallel safety and efficacy study of 2%pirenzepineophthalmicgelinchildrenwithmyopia. *J. Am. Assoc. Pediatr. Ophthalmol. Strabismus* 12, 332–339 (2008).

36. Tan, D. T. H. *et al.* One-year multicenter, double-masked, placebo-controlled, parallel safety and efficacy study of 2% pirenzepine ophthalmic gel in children with myopia. *Ophthalmology* 112, 84–91 (2005).

37. Walline, J. J. *et al.* The Children’s Overnight Orthokeratology Investigation (COOKI) pilot study. *Optom. Vis. Sci.* 81, 407–413 (2004).

38. Walline, J. J. *et al.* Randomized trial of the effect of contact lens wear on self-perception in children. *Optom. Vis. Sci.* 86, 222–232 (2009).

39. Walline, J. J. *et al.* Multifocal contact lens myopia control. *Optom. Vis. Sci.* 90, 1207–1214 (2013).

40. Wu, P.-C. *et al.* The long-term results of using low-concentration atropine eye drops for controlling myopia progression in schoolchildren. *J. Ocul. Pharmacol. Ther.* 27, 461–466 (2011).

41. Yang, Z. *et al.* The effectiveness of progressive addition lenses on the progression of myopia in Chinese children. *Ophthalmic Physiol. Opt.* 29, 41–48 (2009).

42. Yen, M.-Y., Lru, J.-H., Kao, S.-C. & Shiao, C.-H. Comparison of the Effect of Atropine and Cyciopentoiate on Myopia. *Ann* 21, 180–187 (1989).

43. Yi, S. *et al.* Therapeutic effect of atropine 1% in children with low myopia. *J. Am. Assoc. Pediatr. Ophthalmol. Strabismus* 19, 426–429 (2015).

44. Zhu, M.-J., Feng, H.-Y., He, X.-G., Zou, H.-D. & Zhu, J.-F. The control effect of orthokeratology on axial length elongation in Chinese children with myopia. *BMC Ophthalmol.* 14, 141 (2014).

**Appendix 4**

**Forest plots of comparisons**

***
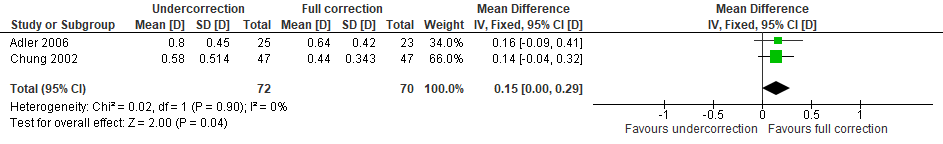
****Undercorrected vs fully-corrected spectacles.* Primary outcomes from baseline (1 year) – Change in refractive error (D).

***
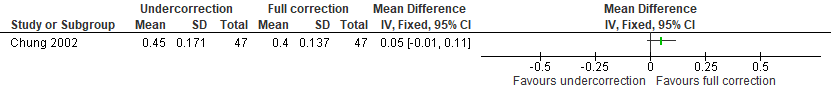
****Undercorrected vs fully-corrected spectacles.* Primary outcomes from baseline (1 year) – Change in axial length (mm).

***
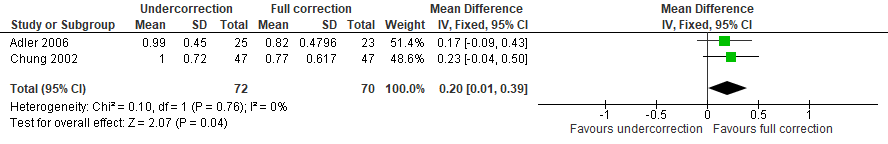
****Undercorrected vs fully-corrected spectacles.* Primary outcomes from baseline (2 years) – Change in refractive error (D).


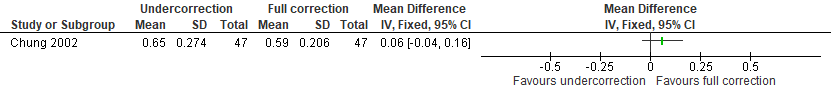
*Undercorrected vs fully-corrected spectacles.* Primary outcomes from baseline (2 years) – Change in axial length (mm).

***
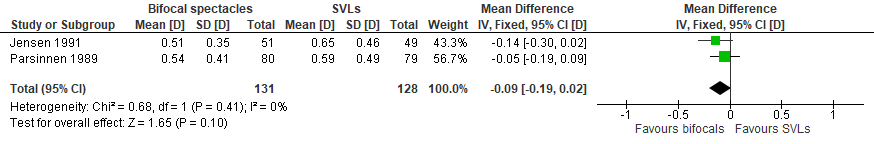
***

*Bifocal spectacles vs single vision lens spectacles (SVLs).* Primary outcomes from baseline (1 year) – Change in refractive error (D).

***
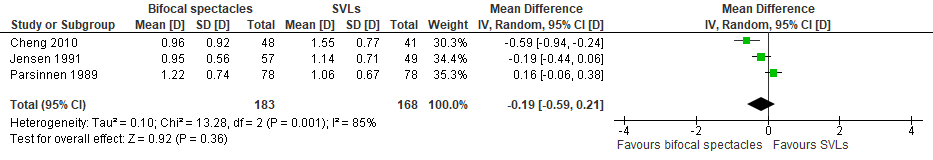
***

*Bifocal spectacles vs single vision lens spectacles (SVLs).* Primary outcomes from baseline (2 years) – Change in refractive error (D).


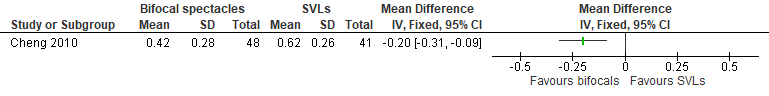
*Bifocal spectacles vs single vision lens spectacles (SVLs).* Primary outcomes from baseline (2 years) – Change in axial length (mm).

***
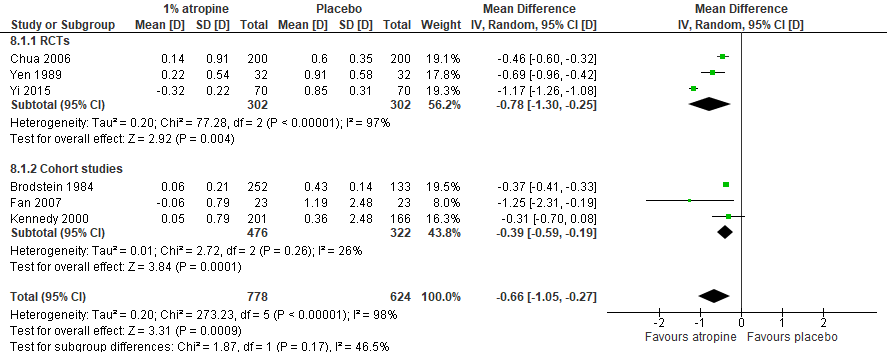
***

*1% Atropine vs placebo.* Primary outcomes from baseline (1 year) – Change in refractive error (D).

***
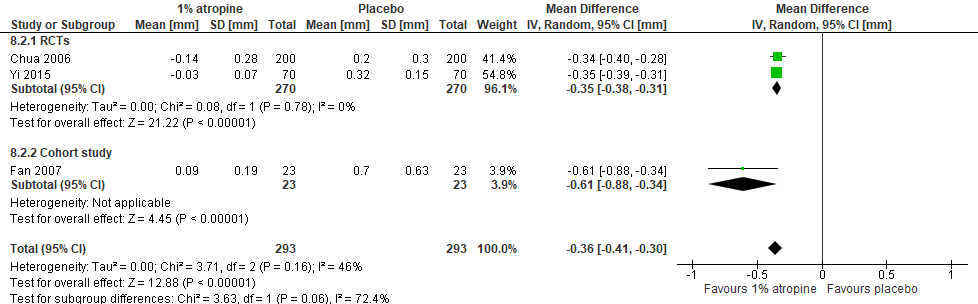
***

*1% Atropine vs placebo.* Primary outcomes from baseline (1 year) – Change in axial length (mm).

*
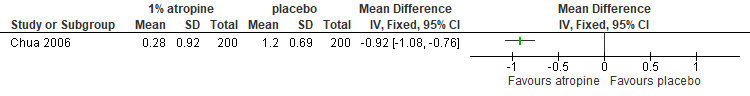
1% Atropine vs placebo.* Primary outcomes from baseline (2 years) – Change in refractive error (D).

*
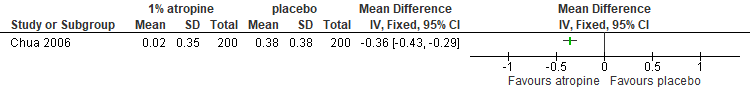
1% Atropine vs placebo.* Primary outcomes from baseline (2 years) – Change in axial length (mm).

12.

*
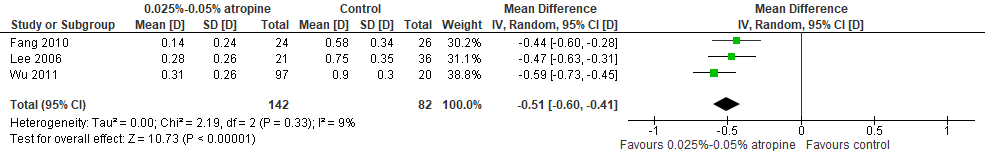
0.025% to 0.05% Atropine vs control.* Primary outcomes from baseline (1 year) – Change in refractive error (D).

13.

*
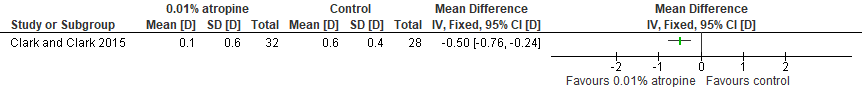
0.01% Atropine vs control.* Primary outcomes from baseline (1 year) – Change in refractive error (D).

14.

*
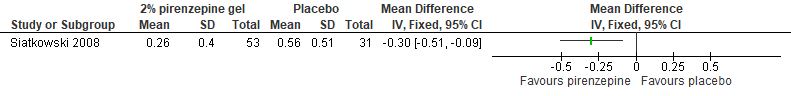
2% Pirenzepine gel vs placebo.* Primary outcomes from baseline (1 year) – Change in refractive error (D).

15.

***
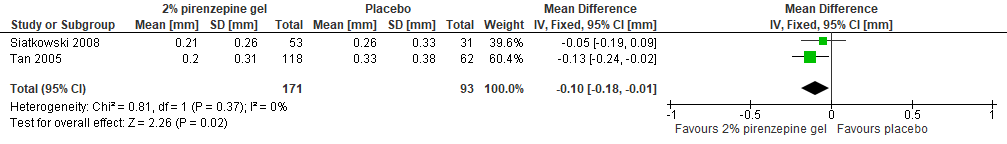
****2% Pirenzepine gel vs placebo.* Primary outcomes from baseline (1 year) – Change in axial length (mm).

16.

*
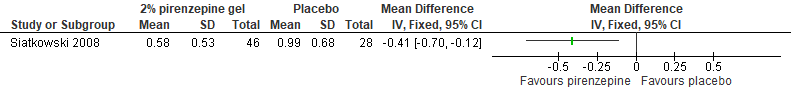
2% Pirenzepine gel vs placebo.* Primary outcomes from baseline (2 years) – Change in refractive error (D).

17.


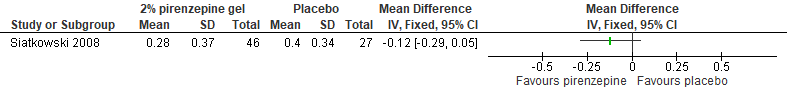
*2% Pirenzepine gel vs placebo.* Primary outcomes from baseline (2 years) – Change in axial length (mm).

18.

***
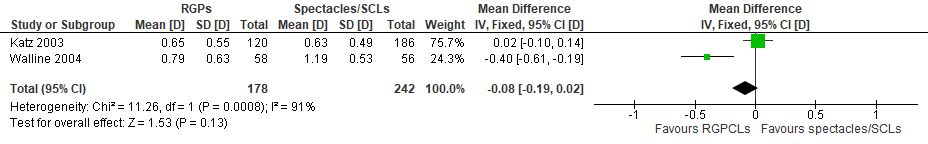
***

*Rigid gas permeable contact lenses (RGPCLs) vs spectacles/soft contact lenses (SCLs).* Primary outcomes from baseline (1 year) – Change in refractive error (D).

19.

***
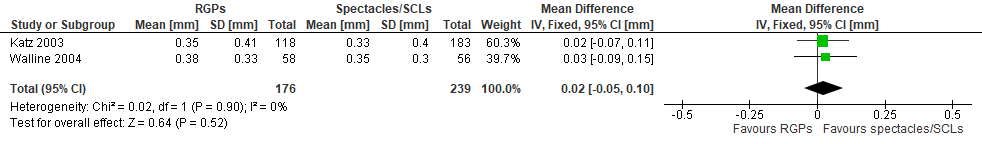
***

*Rigid gas permeable contact lenses (RGPCLs) vs spectacles/soft contact lenses (SCLs).* Primary outcomes from baseline (1 year) – Change in axial length (mm).

20.

***
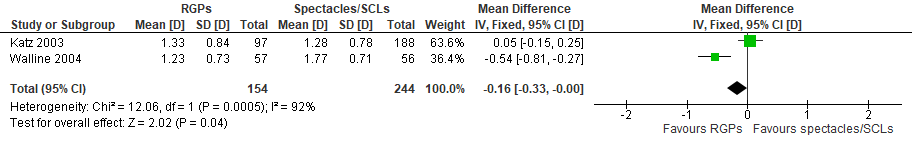
***

*Rigid gas permeable contact lenses (RGPCLs) vs spectacles/soft contact lenses (SCLs).* Primary outcomes from baseline (2 years) – Change in refractive error (D).

21.


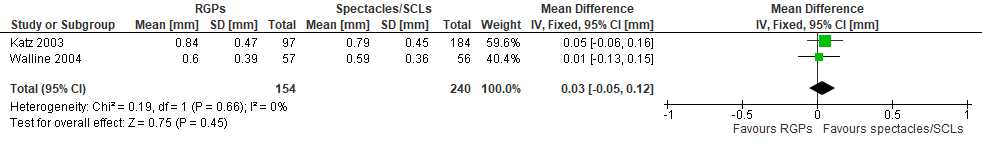


*Rigid gas permeable contact lenses (RGPCLs) vs spectacles/soft contact lenses (SCLs).* Primary outcomes from baseline (2 years) – Change in axial length (mm).

22.

***
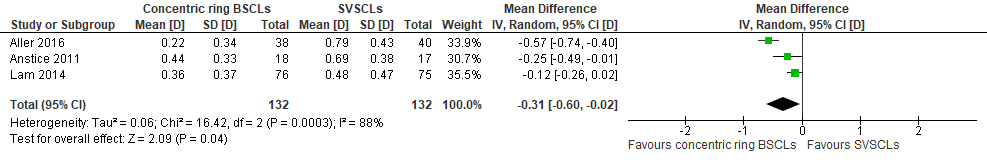
***

*Concentric ring bifocal soft contact lenses (BSCLs) vs single vision soft contact lenses (SVSCLs).* Primary outcomes from baseline (1 year) – Change in refractive error (D).

23.

***
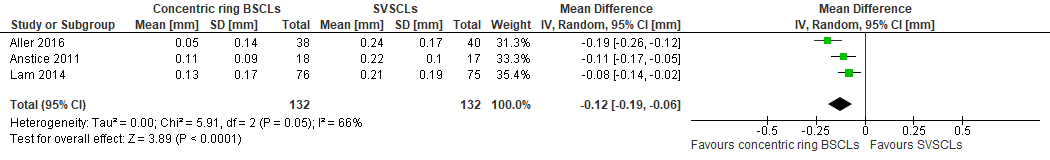
***

*Concentric ring bifocal soft contact lenses (BSCLs) vs single vision soft contact lenses (SVSCLs).* Primary outcomes from baseline (1 year) – Change in axial length (mm).

24.

*
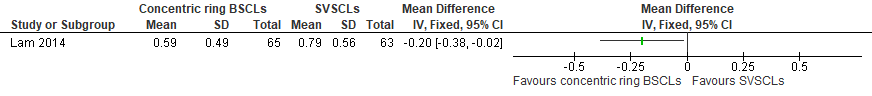
*

*Concentric ring bifocal soft contact lenses (BSCLs) vs single vision soft contact lenses (SVSCLs).* Primary outcomes from baseline (2 years) – Change in refractive error (D).

25.

*
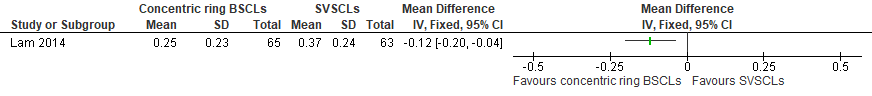
*

*Concentric ring bifocal soft contact lenses (BSCLs) vs single vision soft contact lenses (SVSCLs).* Primary outcomes from baseline (2 years) – Change in axial length (mm).

26.

***
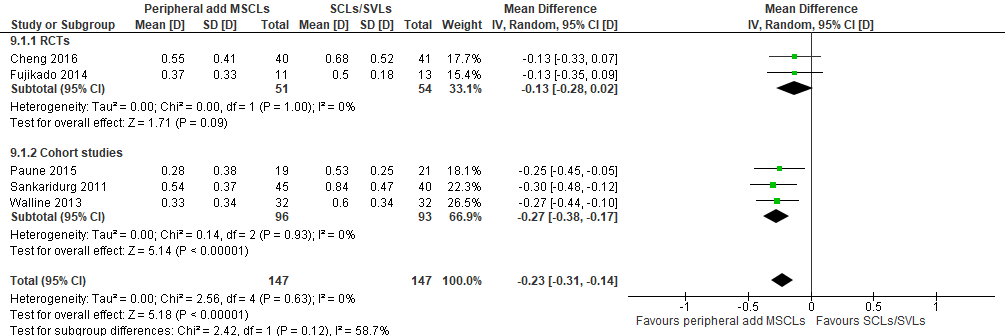
***

*Peripheral add multifocal soft contact lenses (SCLs) vs soft contact lenses (SCLs)/single vision lenses (SVLs).* Primary outcomes from baseline (1 year) – Change in refractive error (D).

27.

***
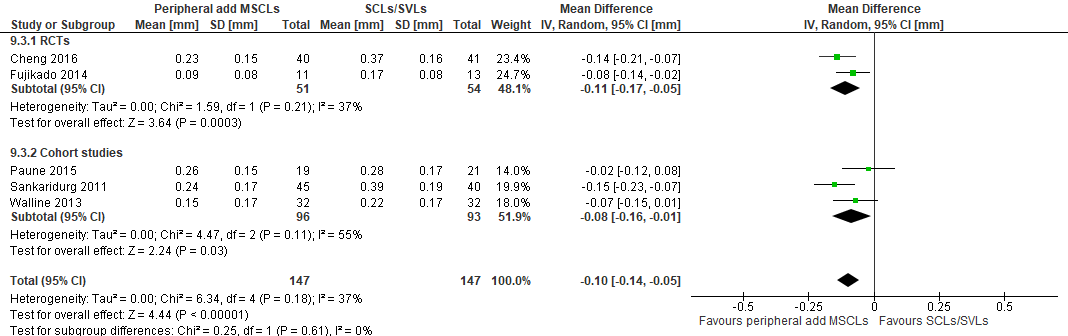
***

*Peripheral add multifocal soft contact lenses (SCLs) vs soft contact lenses (SCLs)/single vision lenses (SVLs).* Primary outcomes from baseline (1 year) – Change in axial length (mm).

28.

***
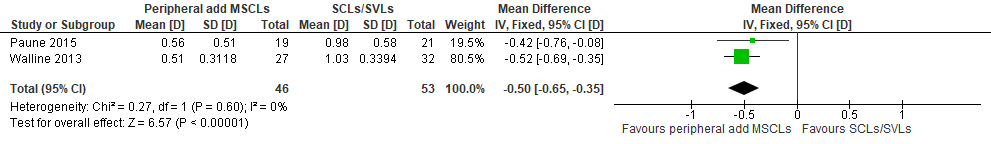
***

*Peripheral add multifocal soft contact lenses (SCLs) vs soft contact lenses (SCLs)/single vision lenses (SVLs).* Primary outcomes from baseline (2 years) – Change in refractive error (D).

29.

***
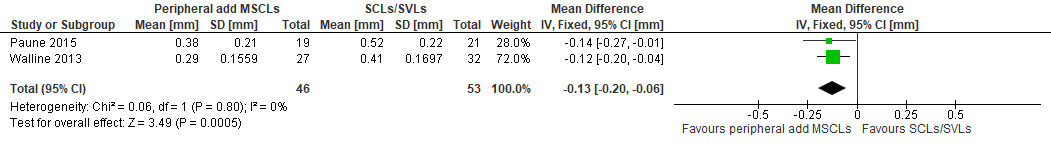
***

*Peripheral add multifocal soft contact lenses (SCLs) vs soft contact lenses (SCLs)/single vision lenses (SVLs).* Primary outcomes from baseline (2 years) – Change in axial length (mm).

30.

*
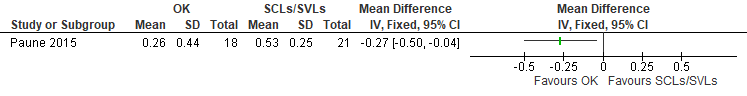
Orthokeratology (OK) vs soft contact lenses (SCLs)/single vision lenses (SVLs).* Primary outcomes from baseline (1 year) – Change in refractive error (D).

31.

***
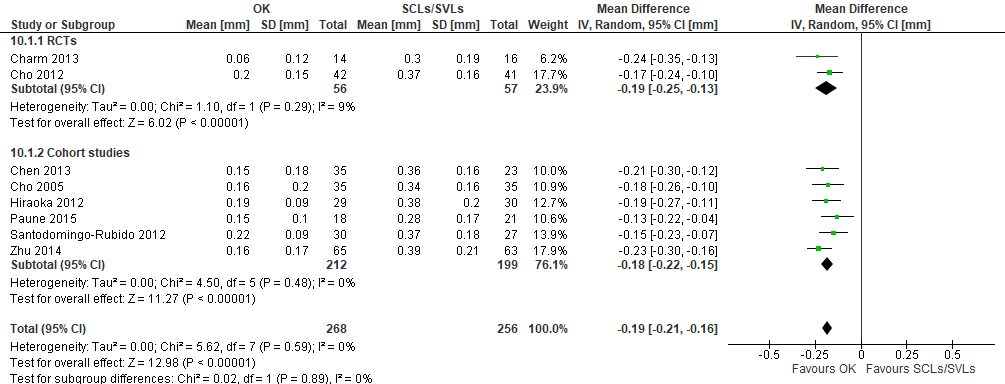
***

*Orthokeratology (OK) vs soft contact lenses (SCLs)/single vision lenses (SVLs).* Primary outcomes from baseline (1 year) – Change in axial length (mm).

32.


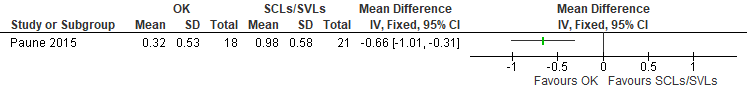
*Orthokeratology (OK) vs soft contact lenses (SCLs)/single vision lenses (SVLs).* Primary outcomes from baseline (2 years) – Change in refractive error (D).

33.

***
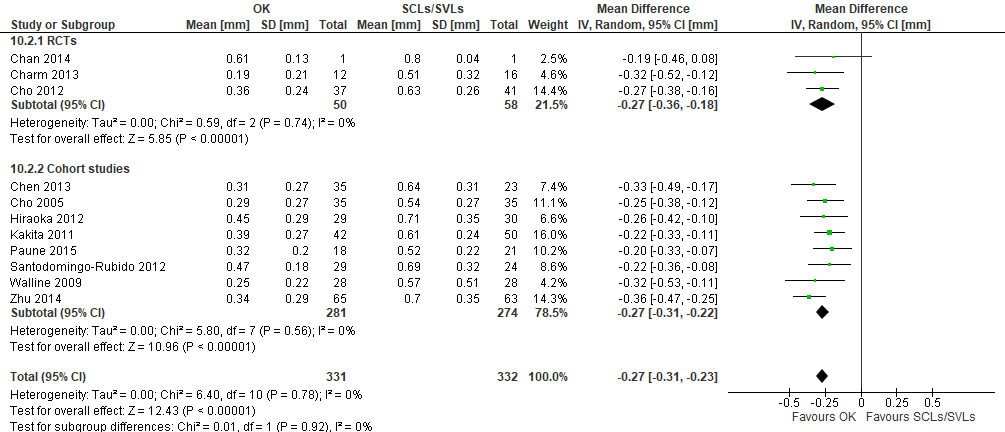
***

*Orthokeratology (OK) vs soft contact lenses (SCLs)/single vision lenses (SVLs).* Primary outcomes from baseline (2 years) – Change in axial length (mm).

34.

***
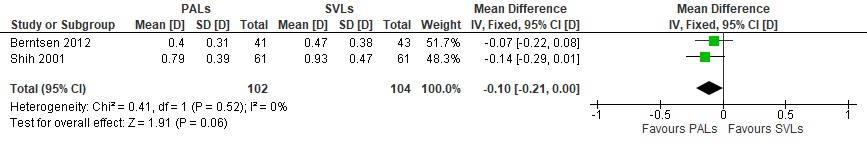
***

*Progressive addition lenses (PALs) vs single vision lenses (SVLs).* Primary outcomes from baseline (1 year) – Change in refractive error (D).

35.

***
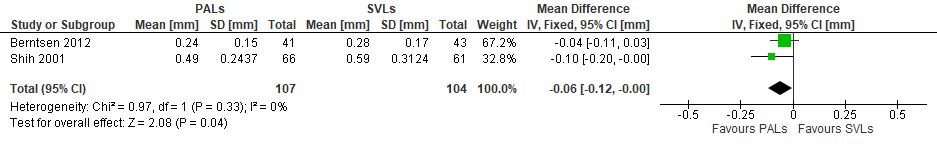
***

*Progressive addition lenses (PALs) vs single vision lenses (SVLs).* Primary outcomes from baseline (1 year) – Change in axial length (mm).

36.


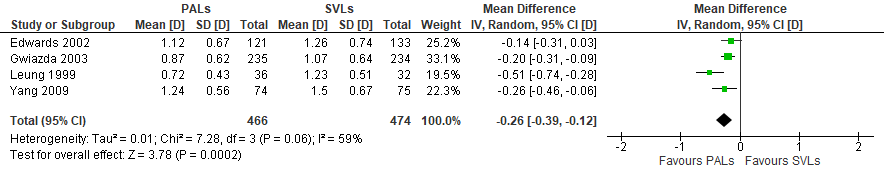


*Progressive addition lenses (PALs) vs single vision lenses (SVLs).* Primary outcomes from baseline (2 years) – Change in refractive error (D).

37.

***
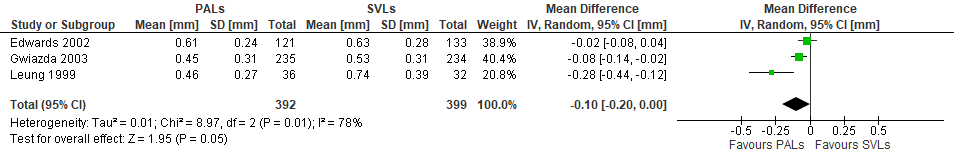
***

*Progressive addition lenses (PALs) vs single vision lenses (SVLs).* Primary outcomes from baseline (2 years) – Change in axial length (mm).

**Appendix 5**

**Forest plots of adverse events**

***
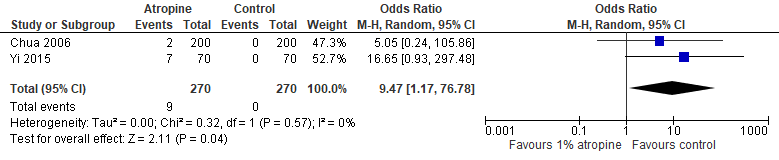
***

*1% Atropine vs placebo.* Blurred near vision.

***
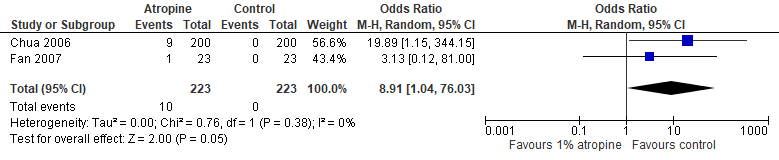
***

*1% Atropine vs placebo.* Allergic or hypersensitivity reactions or discomfort.

***
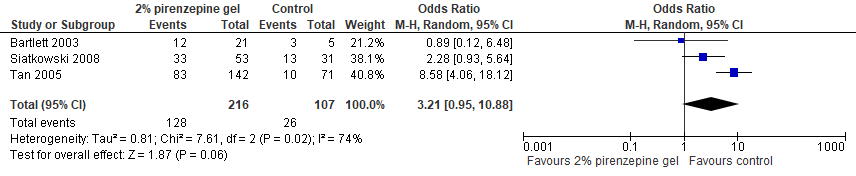
***

*Pirenzepine.* Papillae/Follicles.

***
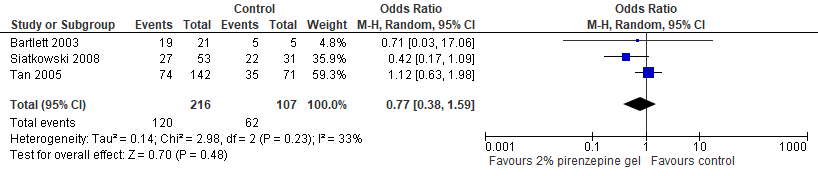
***

*Pirenzepine.* Medication residue on eyelids or eye.

***
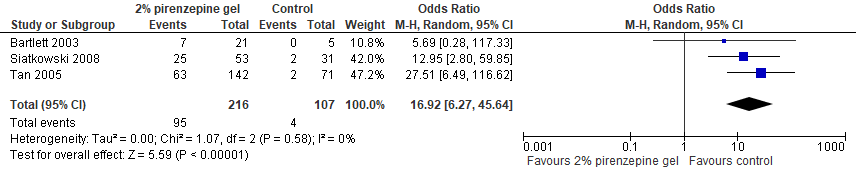
***

*Pirenzepine.* Abnormality of accommodation.

***
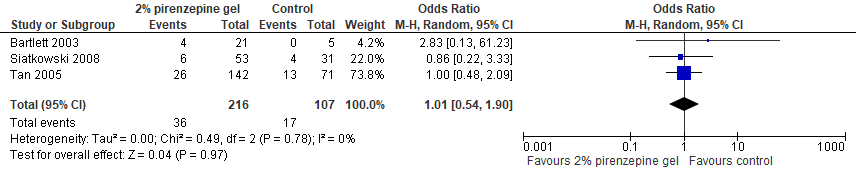
***

*Pirenzepine.* Itching, eye.

***
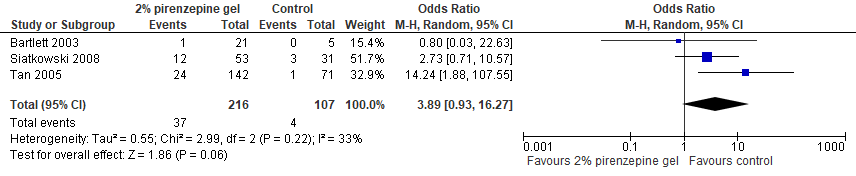
***

*Pirenzepine.* Visual acuity decreased (subjectively).

***
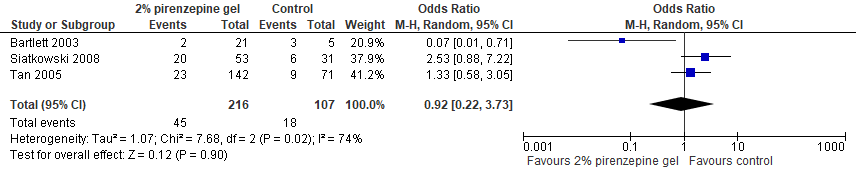
***

*Pirenzepine.* Injection.

***
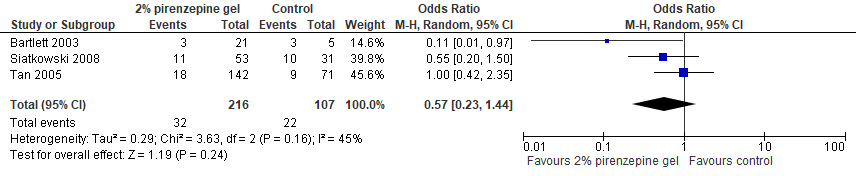
***

*Pirenzepine.* Fluorescein staining.

***
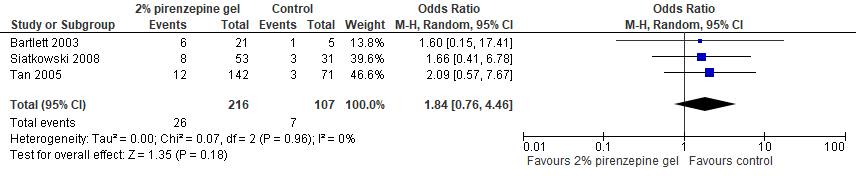
***

*Pirenzepine.* Burn/Sting, eye, on instillation.

***
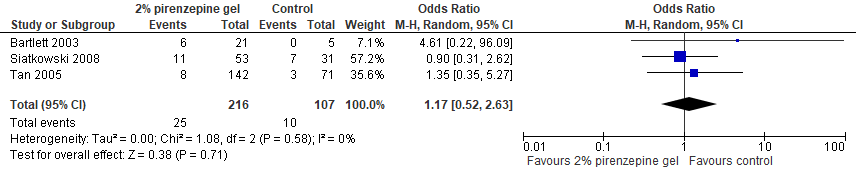
***

*Pirenzepine.* Eye/Vision, blurred.

***
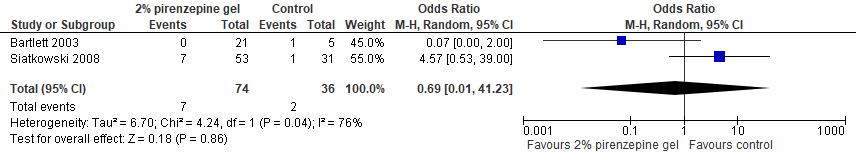
***

*Pirenzepine*. Erythema, eyelids.

***
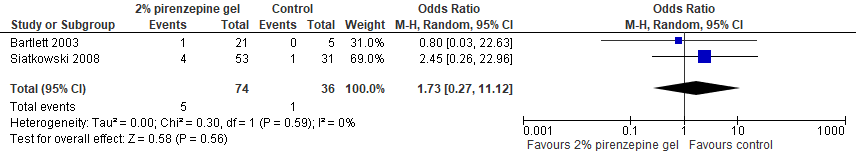
****Pirenzepine.* Eyelid abnormality.

***
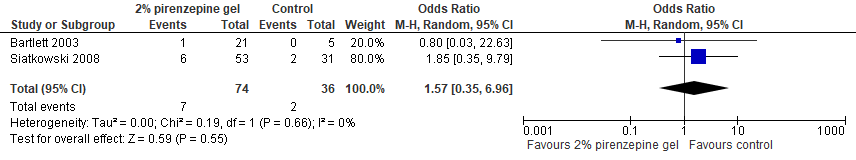
****Pirenzepine.* Photophobia.

***
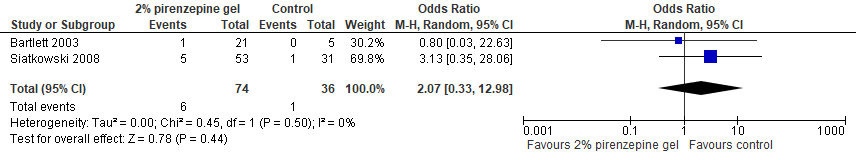
***

*Pirenzepine.* Eye pain.

***
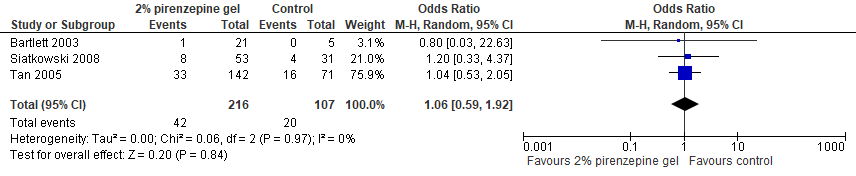
***

*Pirenzepine.* Cough increased.

***
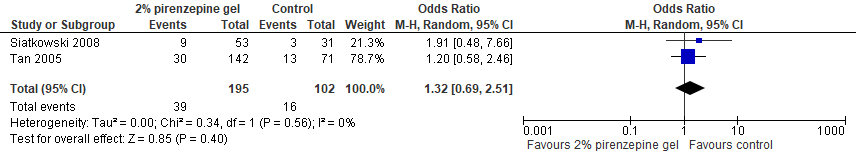
***

*Pirenzepine.* Infection, respiratory.

***
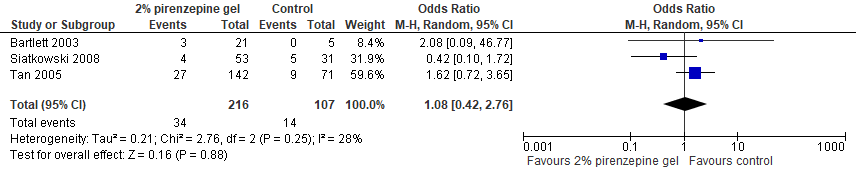
***

*Pirenzepine.* Rhinitis/Sinusitis.

***
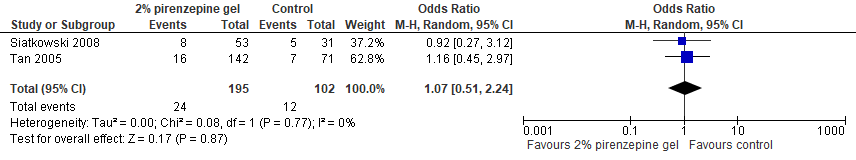
***

*Pirenzepine.* Fever.

***
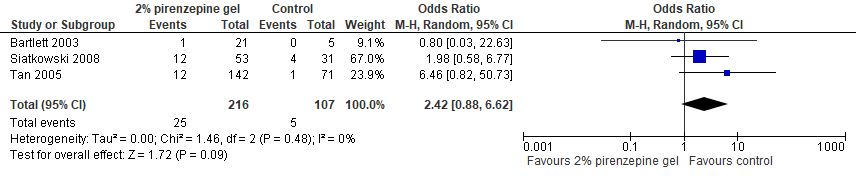
***

*Pirenzepine.* Abdominal pain.

***
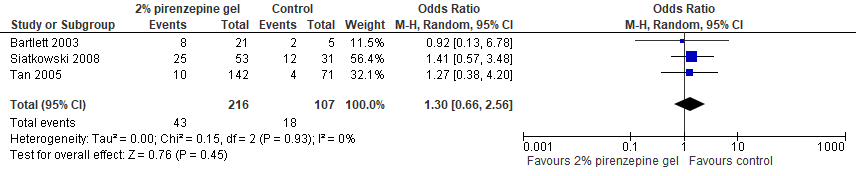
***

*Pirenzepine.* Headache.

***
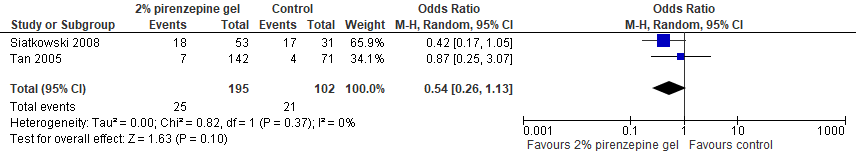
***

*Pirenzepine.* Flu syndrome.

***
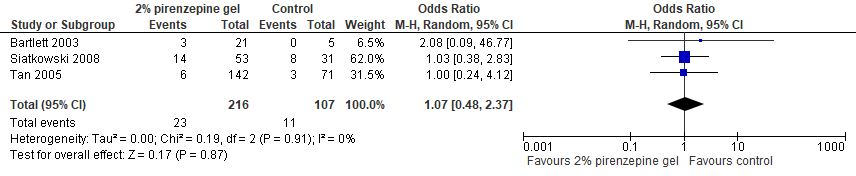
***

*Pirenzepine.* Pharyngitis.

***
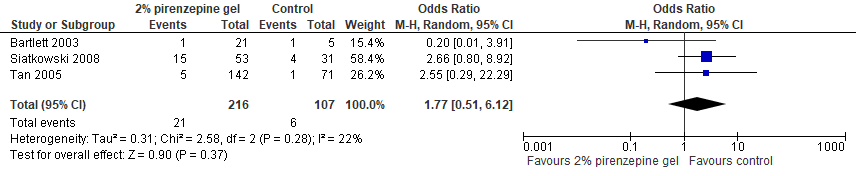
***

*Pirenzepine.* Rash/Allergic reaction.

***
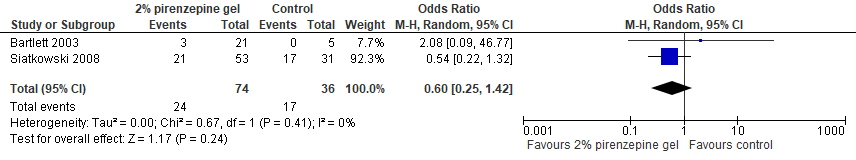
***

*Pirenzepine.* Cold, common.

***
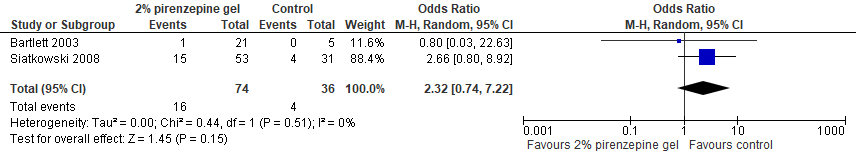
***

*Pirenzepine.* Accidental injury.

***
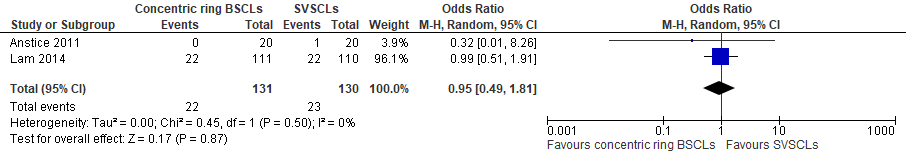
***

*Concentric ring bifocal soft contact lenses.* Contact lens-related discomfort/Unwillingness to wear contact lenses.

***
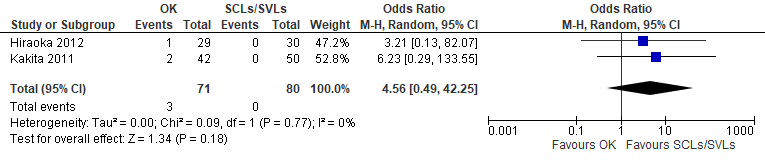
***

*Orthokeratology.* Mild corneal erosion.

**Appendix 6**

**GRADE Assessment of Overall Strength of Evidence**

**Outcome of interest**: Undercorrected compared to fully-corrected spectacles for myopia control

| **Certainty assessment** | | | | | | | **№ of patients** | | **Effect** | | **Certainty** | **Importance** |
| --- | --- | --- | --- | --- | --- | --- | --- | --- | --- | --- | --- | --- |
| **№ of studies** | **Study design** | **Risk of bias** | **Inconsistency** | **Indirectness** | **Imprecision** | **Other considerations** | **Undercorrected** | **fully-corrected spectacles** | **Relative (95% CI)** | **Absolute (95% CI)** |  |  |
| Change in refractive error from baseline (1 year) | | | | | | | | | | | | |
| 2 | randomised trials | not serious | not serious | not serious | serious ^a^ | none | 72 | 70 | - | MD **0.15 higher** (0 to 0.29 higher) | ⨁⨁⨁◯ MODERATE | IMPORTANT |
| Change in refractive error from baseline (2 years) | | | | | | | | | | | | |
| 2 | randomised trials | not serious | not serious | not serious | serious ^b^ | none | 72 | 70 | - | MD **0.2 higher** (0.01 higher to 0.39 higher) | ⨁⨁⨁◯ MODERATE | IMPORTANT |
| Change in axial length from baseline (1 year) | | | | | | | | | | | | |
| 1 | randomised trials | serious ^c^ | serious ^c^ | not serious | very serious ^b^ | none | 47 | 47 | - | MD **0.05 higher** (0.01 lower to 0.11 higher) | ⨁◯◯◯ VERY LOW | NOT IMPORTANT |
| Change in axial length from baseline (2 years) | | | | | | | | | | | | |
| 1 | randomised trials | serious ^c^ | serious ^c^ | not serious | very serious ^b^ | none | 47 | 47 | - | MD **0.06 higher** (0.04 lower to 0.16 higher) | ⨁◯◯◯ VERY LOW | NOT IMPORTANT |

**CI:** Confidence interval; **MD:** Mean difference

**Explanations**

a. CI overlaps no effect with small sample size (<400 participants)

b. Small sample size (<400 participants)

c. Outcome investigated in a single study

**Outcome of interest**: Bifocal spectacles compared to single vision lens spectacles for myopia control

| **Certainty assessment** | | | | | | | **№ of patients** | | **Effect** | | **Certainty** | **Importance** |
| --- | --- | --- | --- | --- | --- | --- | --- | --- | --- | --- | --- | --- |
| **№ of studies** | **Study design** | **Risk of bias** | **Inconsistency** | **Indirectness** | **Imprecision** | **Other considerations** | **Bifocal spectacles** | **Single vision lens spectacles** | **Relative (95% CI)** | **Absolute (95% CI)** |  |  |
| Change in refractive error from baseline (1 year) | | | | | | | | | | | | |
| 2 | randomised trials | not serious | not serious | not serious | serious ^a^ | none | 131 | 128 | - | MD **0.09 lower** (0.19 lower to 0.02 higher) | ⨁⨁⨁◯ MODERATE | IMPORTANT |
| Change in refractive error from baseline (2 years) | | | | | | | | | | | | |
| 3 | randomised trials | not serious | serious ^b^ | not serious | serious ^c^ | none | 183 | 168 | - | MD **0.19 lower** (0.59 lower to 0.21 higher) | ⨁⨁◯◯ LOW | NOT IMPORTANT |
| Change in axial length from baseline (2 years) | | | | | | | | | | | | |
| 1 | randomised trials | serious ^d^ | serious ^d^ | not serious | very serious ^a^ | none | 48 | 41 | - | MD **0.2 lower** (0.31 lower to 0.09 lower) | ⨁◯◯◯ VERY LOW | NOT IMPORTANT |

**CI:** Confidence interval; **MD:** Mean difference

**Explanations**

a. Small sample size (<400 participants)

b. Important, high I^2^

c. CI overlaps no effect with small sample size (<400 participants)

d. Outcome investigated in a single study

**Outcome of interest**: 1% atropine compared to placebo or control for myopia control

| **Certainty assessment** | | | | | | | **№ of patients** | | **Effect** | | **Certainty** | **Importance** |
| --- | --- | --- | --- | --- | --- | --- | --- | --- | --- | --- | --- | --- |
| **№ of studies** | **Study design** | **Risk of bias** | **Inconsistency** | **Indirectness** | **Imprecision** | **Other considerations** | **1% atropine** | **placebo** | **Relative (95% CI)** | **Absolute (95% CI)** |  |  |
| Change in refractive error from baseline (1 year) | | | | | | | | | | | | |
| 6 | randomised trials ^a^ | serious ^b^ | serious ^c^ | not serious | not serious | none | 778 | 624 | - | MD **0.66 lower** (1.05 lower to 0.27 lower) | ⨁⨁◯◯ LOW | IMPORTANT |
| Change in refractive error from baseline (1 year) - RCTs | | | | | | | | | | | | |
| 3 | randomised trials | not serious | serious ^c^ | not serious | not serious | none | 302 | 302 | - | MD **0.78 lower** (1.3 lower to 0.25 lower) | ⨁⨁⨁◯ MODERATE | CRITICAL |
| Change in refractive error from baseline (1 year) - Cohort studies | | | | | | | | | | | | |
| 3 | observational studies | serious ^d^ | not serious | not serious | not serious | none | 476 | 322 | - | MD **0.39 lower** (0.59 lower to 0.19 lower) | ⨁◯◯◯ VERY LOW | NOT IMPORTANT |
| Change in axial length from baseline (1 year) | | | | | | | | | | | | |
| 3 | randomised trials ^a^ | serious ^b^ | not serious | not serious | not serious | none | 293 | 293 | - | MD **0.36 lower** (0.41 lower to 0.3 lower) | ⨁⨁⨁◯ MODERATE | IMPORTANT |
| Change in axial length from baseline (1 year) - RCTs | | | | | | | | | | | | |
| 2 | randomised trials | not serious | not serious | not serious | not serious | none | 270 | 270 | - | MD **0.35 lower** (0.38 lower to 0.31 lower) | ⨁⨁⨁⨁ HIGH | IMPORTANT |
| Change in axial length from baseline (1 year) - Cohort study | | | | | | | | | | | | |
| 1 | observational studies | serious ^e^ | serious ^e^ | not serious | very serious ^f^ | none | 23 | 23 | - | MD **0.61 lower** (0.88 lower to 0.34 lower) | ⨁◯◯◯ VERY LOW | NOT IMPORTANT |
| Allergic or hypersensitivity reactions or discomfort | | | | | | | | | | | | |
| 2 | randomised trials | not serious | not serious | not serious | serious ^g^ | none | 10/223 (4.5%) | 0/223 (0.0%) | **OR 8.91** (1.04 to 76.03) | **0 fewer per 1.000** (from 0 fewer to 0 fewer) | ⨁⨁⨁◯ MODERATE | IMPORTANT |
| Blurred near vision | | | | | | | | | | | | |
| 2 | randomised trials | not serious | not serious | not serious | serious ^g^ | none | 9/270 (3.3%) | 0/270 (0.0%) | **OR 9.47** (1.17 to 76.78) | **0 fewer per 1.000** (from 0 fewer to 0 fewer) | ⨁⨁⨁◯ MODERATE | IMPORTANT |
| Change in refractive error from baseline (2 years) | | | | | | | | | | | | |
| 1 | randomised trials | serious ^e^ | serious ^e^ | not serious | not serious | none | 200 | 200 | - | MD **0.92 lower** (1.08 lower to 0.76 lower) | ⨁⨁◯◯ LOW | IMPORTANT |
| Change in axial length from baseline (2 years) | | | | | | | | | | | | |
| 1 | randomised trials | serious ^e^ | serious ^e^ | not serious | not serious | none | 200 | 200 | - | MD **0.36 lower** (0.43 lower to 0.29 lower) | ⨁⨁◯◯ LOW | IMPORTANT |

**CI:** Confidence interval; **MD:** Mean difference; **OR:** Odds ratio

**Explanations**

a. Randomised trials & observational studies

b. Pooled estimate encompasses data from randomised trials and observational studies

c. Important, high I^2^

d. Observational studies; lower level of evidence

e. Outcome investigated in a single study

f. Small sample size (<400 participants)

g. Wide CIs; imprecision around effect estimate

**Outcome of interest**: 0.025% and 0.05% atropine compared to control for myopia control

| **Certainty assessment** | | | | | | | **№ of patients** | | **Effect** | | **Certainty** | **Importance** |
| --- | --- | --- | --- | --- | --- | --- | --- | --- | --- | --- | --- | --- |
| **№ of studies** | **Study design** | **Risk of bias** | **Inconsistency** | **Indirectness** | **Imprecision** | **Other considerations** | **0.025% and 0.05% atropine** | **control** | **Relative (95% CI)** | **Absolute (95% CI)** |  |  |
| Change in refractive error from baseline (1 year) | | | | | | | | | | | | |
| 3 | observational studies | serious ^a^ | not serious | not serious | serious ^b^ | none | 142 | 82 | - | MD **0.51 lower** (0.6 lower to 0.41 lower) | ⨁◯◯◯ VERY LOW | IMPORTANT |

**CI:** Confidence interval; **MD:** Mean difference

**Explanations**

a. Observational studies; lower level of evidence

b. Small sample size (<400 participants)

**Outcome of interest**: 0.01% atropine compared to control for myopia control

| **Certainty assessment** | | | | | | | **№ of patients** | | **Effect** | | **Certainty** | **Importance** |
| --- | --- | --- | --- | --- | --- | --- | --- | --- | --- | --- | --- | --- |
| **№ of studies** | **Study design** | **Risk of bias** | **Inconsistency** | **Indirectness** | **Imprecision** | **Other considerations** | **0.01% atropine** | **control** | **Relative (95% CI)** | **Absolute (95% CI)** |  |  |
| Change in refractive error from baseline (1 year) | | | | | | | | | | | | |
| 1 | observational studies | very serious ^a,b^ | serious ^a^ | not serious | serious ^c^ | none | 32 | 28 | - | MD **0.5 lower** (0.76 lower to 0.24 lower) | ⨁◯◯◯ VERY LOW | IMPORTANT |

**CI:** Confidence interval; **MD:** Mean difference

**Explanations**

a. Outcome investigated in a single study

b. Observational study; lower level of evidence

c. Small sample size (<400 participants)

**Outcome of interest**: 2% pirenzepine gel compared to placebo for myopia control

| **Certainty assessment** | | | | | | | **№ of patients** | | **Effect** | | **Certainty** | **Importance** |
| --- | --- | --- | --- | --- | --- | --- | --- | --- | --- | --- | --- | --- |
| **№ of studies** | **Study design** | **Risk of bias** | **Inconsistency** | **Indirectness** | **Imprecision** | **Other considerations** | **2% pirenzepine gel** | **placebo** | **Relative (95% CI)** | **Absolute (95% CI)** |  |  |
| Change in refractive error from baseline (1 year) | | | | | | | | | | | | |
| 1 | randomised trials | serious ^a^ | serious ^a^ | not serious | serious ^b^ | none | 53 | 31 | - | MD **0.3 lower** (0.51 lower to 0.09 lower) | ⨁◯◯◯ VERY LOW | NOT IMPORTANT |
| Change in axial length from baseline (1 year) | | | | | | | | | | | | |
| 2 | randomised trials | not serious | not serious | not serious | serious ^b^ | none | 171 | 93 | - | MD **0.1 lower** (0.18 lower to 0.01 lower) | ⨁⨁⨁◯ MODERATE | IMPORTANT |
| Papillae/Follicles | | | | | | | | | | | | |
| 3 | randomised trials | not serious | serious ^c^ | not serious | serious ^d^ | none | 128/216 (59.3%) | 26/107 (24.3%) | **OR 3.21** (0.95 to 10.88) | **264 more per 1.000** (from 9 fewer to 534 more) | ⨁⨁◯◯ LOW | NOT IMPORTANT |
| Medication residue on eyelids or eye | | | | | | | | | | | | |
| 3 | randomised trials | not serious | not serious | not serious | serious ^d^ | none | 120/216 (55.6%) | 62/107 (57.9%) | **OR 0.77** (0.38 to 1.59) | **65 fewer per 1.000** (from 107 more to 236 fewer) | ⨁⨁⨁◯ MODERATE | NOT IMPORTANT |
| Abnormality of accommodation | | | | | | | | | | | | |
| 3 | randomised trials | not serious | not serious | not serious | serious ^b,e^ | none | 95/216 (44.0%) | 4/107 (3.7%) | **OR 16.92** (6.27 to 45.64) | **359 more per 1.000** (from 158 more to 602 more) | ⨁⨁⨁◯ MODERATE | NOT IMPORTANT |
| Itching, eye | | | | | | | | | | | | |
| 3 | randomised trials | not serious | not serious | not serious | serious ^d^ | none | 36/216 (16.7%) | 17/107 (15.9%) | **OR 1.01** (0.54 to 1.90) | **1 more per 1.000** (from 66 fewer to 105 more) | ⨁⨁⨁◯ MODERATE | NOT IMPORTANT |
| Visual acuity decreased (subjectively) | | | | | | | | | | | | |
| 3 | randomised trials | not serious | not serious | not serious | serious ^d^ | none | 37/216 (17.1%) | 4/107 (3.7%) | **OR 3.89** (0.93 to 16.27) | **94 more per 1.000** (from 3 fewer to 350 more) | ⨁⨁⨁◯ MODERATE | NOT IMPORTANT |
| Injection | | | | | | | | | | | | |
| 3 | randomised trials | not serious | serious ^c^ | not serious | serious ^d^ | none | 45/216 (20.8%) | 18/107 (16.8%) | **OR 0.92** (0.22 to 3.73) | **11 fewer per 1.000** (from 126 fewer to 262 more) | ⨁⨁◯◯ LOW | NOT IMPORTANT |
| Fluorecein staining | | | | | | | | | | | | |
| 3 | randomised trials | not serious | not serious | not serious | serious ^d^ | none | 32/216 (14.8%) | 22/107 (20.6%) | **OR 0.57** (0.23 to 1.44) | **77 fewer per 1.000** (from 66 more to 149 fewer) | ⨁⨁⨁◯ MODERATE | NOT IMPORTANT |
| Burn/Sting, eye, on instillation | | | | | | | | | | | | |
| 3 | randomised trials | not serious | not serious | not serious | serious ^d^ | none | 26/216 (12.0%) | 7/107 (6.5%) | **OR 1.84** (0.76 to 4.46) | **49 more per 1.000** (from 15 fewer to 173 more) | ⨁⨁⨁◯ MODERATE | NOT IMPORTANT |
| Eye/Vision, blurred | | | | | | | | | | | | |
| 3 | randomised trials | not serious | not serious | not serious | serious ^d^ | none | 25/216 (11.6%) | 10/107 (9.3%) | **OR 1.17** (0.52 to 2.63) | **14 more per 1.000** (from 43 fewer to 120 more) | ⨁⨁⨁◯ MODERATE | NOT IMPORTANT |
| Erythema, eyelids | | | | | | | | | | | | |
| 2 | randomised trials | not serious | serious ^f^ | not serious | very serious ^d,e^ | none | 7/74 (9.5%) | 2/36 (5.6%) | **OR 0.69** (0.01 to 41.23) | **17 fewer per 1.000** (from 55 fewer to 652 more) | ⨁◯◯◯ VERY LOW | NOT IMPORTANT |
| Eyelid abnormality | | | | | | | | | | | | |
| 2 | randomised trials | not serious | not serious | not serious | serious ^d^ | none | 5/74 (6.8%) | 1/36 (2.8%) | **OR 1.73** (0.27 to 11.12) | **19 more per 1.000** (from 20 fewer to 213 more) | ⨁⨁⨁◯ MODERATE | NOT IMPORTANT |
| Photophobia | | | | | | | | | | | | |
| 2 | randomised trials | not serious | not serious | not serious | serious ^d^ | none | 7/74 (9.5%) | 2/36 (5.6%) | **OR 1.57** (0.35 to 6.96) | **29 more per 1.000** (from 35 fewer to 235 more) | ⨁⨁⨁◯ MODERATE | NOT IMPORTANT |
| Eye pain | | | | | | | | | | | | |
| 2 | randomised trials | not serious | not serious | not serious | serious ^d^ | none | 6/74 (8.1%) | 1/36 (2.8%) | **OR 2.07** (0.33 to 12.98) | **28 more per 1.000** (from 18 fewer to 243 more) | ⨁⨁⨁◯ MODERATE | NOT IMPORTANT |
| Cough increased | | | | | | | | | | | | |
| 3 | randomised trials | not serious | not serious | not serious | serious ^d^ | none | 42/216 (19.4%) | 20/107 (18.7%) | **OR 1.06** (0.59 to 1.92) | **9 more per 1.000** (from 67 fewer to 119 more) | ⨁⨁⨁◯ MODERATE | NOT IMPORTANT |
| Infection, respiratory | | | | | | | | | | | | |
| 2 | randomised trials | not serious | not serious | not serious | serious ^d^ | none | 39/195 (20.0%) | 16/102 (15.7%) | **OR 1.32** (0.69 to 2.51) | **40 more per 1.000** (from 43 fewer to 161 more) | ⨁⨁⨁◯ MODERATE | NOT IMPORTANT |
| Rhinitis/Sinusitis | | | | | | | | | | | | |
| 3 | randomised trials | not serious | not serious | not serious | serious ^d^ | none | 34/216 (15.7%) | 14/107 (13.1%) | **OR 1.08** (0.42 to 2.76) | **9 more per 1.000** (from 71 fewer to 163 more) | ⨁⨁⨁◯ MODERATE | NOT IMPORTANT |
| Fever | | | | | | | | | | | | |
| 2 | randomised trials | not serious | not serious | not serious | serious ^d^ | none | 24/195 (12.3%) | 12/102 (11.8%) | **OR 1.07** (0.51 to 2.24) | **7 more per 1.000** (from 54 fewer to 112 more) | ⨁⨁⨁◯ MODERATE | NOT IMPORTANT |
| Abdominal pain | | | | | | | | | | | | |
| 3 | randomised trials | not serious | not serious | not serious | serious ^d^ | none | 25/216 (11.6%) | 5/107 (4.7%) | **OR 2.42** (0.88 to 6.62) | **59 more per 1.000** (from 5 fewer to 198 more) | ⨁⨁⨁◯ MODERATE | NOT IMPORTANT |
| Headache | | | | | | | | | | | | |
| 3 | randomised trials | not serious | not serious | not serious | serious ^d^ | none | 43/216 (19.9%) | 18/107 (16.8%) | **OR 1.30** (0.66 to 2.56) | **40 more per 1.000** (from 50 fewer to 173 more) | ⨁⨁⨁◯ MODERATE | NOT IMPORTANT |
| Flu syndrome | | | | | | | | | | | | |
| 2 | randomised trials | not serious | not serious | not serious | serious ^d^ | none | 25/195 (12.8%) | 21/102 (20.6%) | **OR 0.54** (0.26 to 1.13) | **83 fewer per 1.000** (from 21 more to 143 fewer) | ⨁⨁⨁◯ MODERATE | NOT IMPORTANT |
| Pharyngitis | | | | | | | | | | | | |
| 3 | randomised trials | not serious | not serious | not serious | serious ^d^ | none | 23/216 (10.6%) | 11/107 (10.3%) | **OR 1.07** (0.48 to 2.37) | **6 more per 1.000** (from 51 fewer to 111 more) | ⨁⨁⨁◯ MODERATE | NOT IMPORTANT |
| Rash/Allergic reaction | | | | | | | | | | | | |
| 3 | randomised trials | not serious | not serious | not serious | serious ^d^ | none | 21/216 (9.7%) | 6/107 (5.6%) | **OR 1.77** (0.51 to 6.12) | **39 more per 1.000** (from 27 fewer to 211 more) | ⨁⨁⨁◯ MODERATE | NOT IMPORTANT |
| Cold, common | | | | | | | | | | | | |
| 2 | randomised trials | not serious | not serious | not serious | serious ^d^ | none | 24/74 (32.4%) | 17/36 (47.2%) | **OR 0.60** (0.25 to 1.42) | **123 fewer per 1.000** (from 87 more to 289 fewer) | ⨁⨁⨁◯ MODERATE | NOT IMPORTANT |
| Accidental injury | | | | | | | | | | | | |
| 2 | randomised trials | not serious | not serious | not serious | serious ^d^ | none | 16/74 (21.6%) | 4/36 (11.1%) | **OR 2.32** (0.74 to 7.22) | **114 more per 1.000** (from 26 fewer to 363 more) | ⨁⨁⨁◯ MODERATE | NOT IMPORTANT |
| Change in refractive error from baseline (2 years) | | | | | | | | | | | | |
| 1 | randomised trials | serious ^a^ | serious ^a^ | not serious | serious ^b^ | none | 46 | 28 | - | MD **0.41 lower** (0.7 lower to 0.12 lower) | ⨁◯◯◯ VERY LOW | NOT IMPORTANT |
| Change in axial length from baseline (2 years) | | | | | | | | | | | | |
| 1 | randomised trials | serious ^a^ | serious ^a^ | not serious | very serious ^d^ | none | 46 | 27 | - | MD **0.12 lower** (0.29 lower to 0.05 higher) | ⨁◯◯◯ VERY LOW | NOT IMPORTANT |

**CI:** Confidence interval; **MD:** Mean difference; **OR:** Odds ratio

**Explanations**

a. Outcome investigated in a single study

b. Small sample size (<400 participants)

c. I^2^ in the moderate range

d. CI overlaps no effect with small sample size (<400 participants)

e. Wide CIs; imprecision around effect estimate

f. Important, high I^2^

**Outcome of interest**: Rigid gas permeable contact lenses compared to spectacles or soft contact lenses for myopia control

| **Certainty assessment** | | | | | | | **№ of patients** | | **Effect** | | **Certainty** | **Importance** |
| --- | --- | --- | --- | --- | --- | --- | --- | --- | --- | --- | --- | --- |
| **№ of studies** | **Study design** | **Risk of bias** | **Inconsistency** | **Indirectness** | **Imprecision** | **Other considerations** | **Rigid gas permeable contact lenses** | **spectacles or soft contact lenses** | **Relative (95% CI)** | **Absolute (95% CI)** |  |  |
| Change in refractive error from baseline (1 year) | | | | | | | | | | | | |
| 2 | randomised trials | not serious | serious ^a^ | not serious | serious ^b^ | none | 178 | 242 | - | MD **0.08 lower** (0.19 lower to 0.02 higher) | ⨁⨁◯◯ LOW | NOT IMPORTANT |
| Change in refractive error from baseline (2 years) | | | | | | | | | | | | |
| 2 | randomised trials | not serious | serious ^a^ | not serious | serious ^b^ | none | 154 | 244 | - | MD **0.16 lower** (0.33 lower to 0 ) | ⨁⨁◯◯ LOW | IMPORTANT |
| Change in axial length from baseline (1 year) | | | | | | | | | | | | |
| 2 | randomised trials | not serious | not serious | not serious | serious ^b^ | none | 176 | 239 | - | MD **0.02 higher** (0.05 lower to 0.1 higher) | ⨁⨁⨁◯ MODERATE | NOT IMPORTANT |
| Change in axial length from baseline (2 years) | | | | | | | | | | | | |
| 2 | randomised trials | not serious | not serious | not serious | serious ^b^ | none | 154 | 240 | - | MD **0.03 higher** (0.05 lower to 0.12 higher) | ⨁⨁⨁◯ MODERATE | IMPORTANT |

**CI:** Confidence interval; **MD:** Mean difference

**Explanations**

a. Important, high I^2^

b. CI overlaps no effect

**Outcome of interest**: Concentric ring bifocal soft contact lenses compared to single vision soft contact lenses for myopia control

| **Certainty assessment** | | | | | | | **№ of patients** | | **Effect** | | **Certainty** | **Importance** |
| --- | --- | --- | --- | --- | --- | --- | --- | --- | --- | --- | --- | --- |
| **№ of studies** | **Study design** | **Risk of bias** | **Inconsistency** | **Indirectness** | **Imprecision** | **Other considerations** | **Concentric ring bifocal soft contact lenses** | **single vision soft contact lenses** | **Relative (95% CI)** | **Absolute (95% CI)** |  |  |
| Change in refractive error from baseline (1 year) | | | | | | | | | | | | |
| 2 | randomised trials | not serious | serious ^a^ | not serious | serious ^b^ | none | 94 | 92 | - | MD **0.15 lower** (0.27 lower to 0.03 lower) | ⨁⨁◯◯ LOW | IMPORTANT |
| Change in axial length from baseline (1 year) | | | | | | | | | | | | |
| 3 | randomised trials | not serious | serious ^c^ | not serious | serious ^b^ | none | 132 | 132 | - | MD **0.12 lower** (0.19 lower to 0.06 lower) | ⨁⨁◯◯ LOW | IMPORTANT |
| Contact lens-related discomfort/Unwillingness to wear contact lenses | | | | | | | | | | | | |
| 2 | randomised trials | not serious | not serious | not serious | serious ^d^ | none | 22/131 (16.8%) | 23/130 (17.7%) | **OR 0.95** (0.49 to 1.81) | **7 fewer per 1.000** (from 82 fewer to 103 more) | ⨁⨁⨁◯ MODERATE | IMPORTANT |
| Change in refractive error from baseline (2 years) | | | | | | | | | | | | |
| 1 | randomised trials | serious ^e^ | serious ^e^ | not serious | serious ^b^ | none | 65 | 63 | - | MD **0.2 lower** (0.38 lower to 0.02 lower) | ⨁◯◯◯ VERY LOW | IMPORTANT |
| Change in axial length from baseline (2 years) | | | | | | | | | | | | |
| 1 | randomised trials | serious ^e^ | serious ^e^ | not serious | serious ^b^ | none | 65 | 63 | - | MD **0.12 lower** (0.2 lower to 0.04 lower) | ⨁◯◯◯ VERY LOW | IMPORTANT |

**CI:** Confidence interval; **MD:** Mean difference; **OR:** Odds ratio

**Explanations**

a. Important, high I^2^

b. Small sample size (<400 participants)

c. I^2^ in the moderate range

d. CI overlaps no effect with small sample size (<400 participants)

e. Outcome investigated in a single study

**Outcome of interest**: Peripheral add multifocal soft contact lenses compared to soft contact lenses or single vision lenses for myopia control

| **Certainty assessment** | | | | | | | **№ of patients** | | **Effect** | | **Certainty** | **Importance** |
| --- | --- | --- | --- | --- | --- | --- | --- | --- | --- | --- | --- | --- |
| **№ of studies** | **Study design** | **Risk of bias** | **Inconsistency** | **Indirectness** | **Imprecision** | **Other considerations** | **Peripheral add multifocal soft contact lenses** | **soft contact lenses or single vision lenses** | **Relative (95% CI)** | **Absolute (95% CI)** |  |  |
| Change in refractive error from baseline (1 year) | | | | | | | | | | | | |
| 5 | randomised trials ^a^ | serious ^b^ | serious ^c^ | not serious | serious ^d^ | none | 147 | 147 | - | MD **0.23 lower** (0.31 lower to 0.14 lower) | ⨁◯◯◯ VERY LOW | NOT IMPORTANT |
| Change in refractive error from baseline (1 year) - RCTs | | | | | | | | | | | | |
| 2 | randomised trials | not serious | not serious | not serious | serious ^e^ | none | 51 | 54 | - | MD **0.13 lower** (0.28 lower to 0.02 higher) | ⨁⨁⨁◯ MODERATE | IMPORTANT |
| Change in refractive error from baseline (1 year) - Cohort studies | | | | | | | | | | | | |
| 3 | observational studies | serious ^f^ | not serious | not serious | serious ^d^ | none | 96 | 93 | - | MD **0.27 lower** (0.38 lower to 0.17 lower) | ⨁◯◯◯ VERY LOW | NOT IMPORTANT |
| Change in refractive error from baseline (2 years) | | | | | | | | | | | | |
| 2 | observational studies | serious ^f^ | not serious | not serious | serious ^d^ | none | 46 | 53 | - | MD **0.5 lower** (0.65 lower to 0.35 lower) | ⨁◯◯◯ VERY LOW | NOT IMPORTANT |
| Change in axial length from baseline (1 year) | | | | | | | | | | | | |
| 5 | randomised trials ^a^ | serious ^b^ | not serious | not serious | serious ^d^ | none | 147 | 147 | - | MD **0.1 lower** (0.14 lower to 0.05 lower) | ⨁⨁◯◯ LOW | IMPORTANT |
| Change in axial length from baseline (1 year) - RCTs | | | | | | | | | | | | |
| 2 | randomised trials | not serious | not serious | not serious | serious ^d^ | none | 51 | 54 | - | MD **0.11 lower** (0.17 lower to 0.05 lower) | ⨁⨁⨁◯ MODERATE | IMPORTANT |
| Change in axial length from baseline (1 year) - Cohort studies | | | | | | | | | | | | |
| 3 | observational studies | serious ^f^ | serious ^c^ | not serious | serious ^d^ | none | 96 | 93 | - | MD **0.08 lower** (0.16 lower to 0.01 lower) | ⨁◯◯◯ VERY LOW | NOT IMPORTANT |
| Change in axial length from baseline (2 years) | | | | | | | | | | | | |
| 2 | observational studies | serious ^f^ | not serious | not serious | serious ^d^ | none | 46 | 53 | - | MD **0.13 lower** (0.2 lower to 0.06 lower) | ⨁◯◯◯ VERY LOW | NOT IMPORTANT |

**CI:** Confidence interval; **MD:** Mean difference

**Explanations**

a. Randomised trials & observational studies

b. Pooled estimate encompasses data from randomised trials and observational studies

c. I^2^ in the moderate range

d. Small sample size (<400 participants)

e. CI overlaps no effect with small sample size (<400 participants)

f. Observational study; lower level of evidence

**Outcome of interest**: Orthokeratology compared to soft contact lenses or single vision lenses for myopia control

| **Certainty assessment** | | | | | | | **№ of patients** | | **Effect** | | **Certainty** | **Importance** |
| --- | --- | --- | --- | --- | --- | --- | --- | --- | --- | --- | --- | --- |
| **№ of studies** | **Study design** | **Risk of bias** | **Inconsistency** | **Indirectness** | **Imprecision** | **Other considerations** | **Orthokeratology** | **soft contact lenses or single vision lenses** | **Relative (95% CI)** | **Absolute (95% CI)** |  |  |
| Change in axial length from baseline (1 year) | | | | | | | | | | | | |
| 8 | randomised trials ^a^ | serious ^b^ | not serious | not serious | not serious | none | 268 | 256 | - | MD **0.19 lower** (0.21 lower to 0.16 lower) | ⨁⨁⨁◯ MODERATE | IMPORTANT |
| Change in axial length from baseline (1 year) - RCTs | | | | | | | | | | | | |
| 2 | randomised trials | not serious | not serious | not serious | serious ^c^ | none | 56 | 57 | - | MD **0.19 lower** (0.25 lower to 0.13 lower) | ⨁⨁⨁◯ MODERATE | IMPORTANT |
| Change in axial length from baseline (1 year) - Cohort studies | | | | | | | | | | | | |
| 6 | observational studies | serious ^d^ | not serious | not serious | not serious | none | 212 | 199 | - | MD **0.18 lower** (0.22 lower to 0.15 lower) | ⨁◯◯◯ VERY LOW | NOT IMPORTANT |
| Change in axial length from baseline (2 years) | | | | | | | | | | | | |
| 11 | randomised trials ^a^ | serious ^b^ | not serious | not serious | not serious | none | 331 | 332 | - | MD **0.27 lower** (0.31 lower to 0.23 lower) | ⨁⨁⨁◯ MODERATE | IMPORTANT |
| Change in axial length from baseline (2 years) - RCTs | | | | | | | | | | | | |
| 3 | randomised trials | not serious | not serious | not serious | serious ^c^ | none | 50 | 58 | - | MD **0.27 lower** (0.36 lower to 0.18 lower) | ⨁⨁⨁◯ MODERATE | IMPORTANT |
| Change in axial length from baseline (2 years) - Cohort studies | | | | | | | | | | | | |
| 8 | observational studies | serious ^d^ | not serious | not serious | not serious | none | 281 | 274 | - | MD **0.27 lower** (0.31 lower to 0.22 lower) | ⨁◯◯◯ VERY LOW | NOT IMPORTANT |
| Mild corneal erosion | | | | | | | | | | | | |
| 2 | observational studies | serious ^d^ | not serious | not serious | very serious ^e,f^ | none | 3/71 (4.2%) | 0/80 (0.0%) | **OR 4.56** (0.49 to 42.25) | **0 fewer per 1.000** (from 0 fewer to 0 fewer) | ⨁◯◯◯ VERY LOW | NOT IMPORTANT |
| Change in refractive error from baseline (1 year) | | | | | | | | | | | | |
| 1 | observational studies | very serious ^d,g^ | serious ^g^ | not serious | serious ^c^ | none | 18 | 21 | - | MD **0.27 lower** (0.5 lower to 0.04 lower) | ⨁◯◯◯ VERY LOW | NOT IMPORTANT |
| Change in refractive error from baseline (2 years) | | | | | | | | | | | | |
| 1 | observational studies | very serious ^d,g^ | serious ^g^ | not serious | serious ^c^ | none | 18 | 21 | - | MD **0.66 lower** (1.01 lower to 0.31 lower) | ⨁◯◯◯ VERY LOW | NOT IMPORTANT |

**CI:** Confidence interval; **MD:** Mean difference; **OR:** Odds ratio

**Explanations**

a. Randomised trials & observational studies

b. Pooled estimate encompasses data from randomised trials and observational studies

c. Small sample size (<400 participants)

d. Observational study; lower level of evidence

e. CI overlaps no effect with small sample size (<400 participants)

f. Wide CIs; imprecision around effect estimate

g. Outcome investigated in a single study

**Outcome of interest**: Progressive addition lenses compared to single vision lenses for myopia control

| **Certainty assessment** | | | | | | | **№ of patients** | | **Effect** | | **Certainty** | **Importance** |
| --- | --- | --- | --- | --- | --- | --- | --- | --- | --- | --- | --- | --- |
| **№ of studies** | **Study design** | **Risk of bias** | **Inconsistency** | **Indirectness** | **Imprecision** | **Other considerations** | **Progressive addition lenses** | **Single vision lenses** | **Relative (95% CI)** | **Absolute (95% CI)** |  |  |
| Change in refractive error from baseline (2 years) | | | | | | | | | | | | |
| 4 | randomised trials | not serious | serious ^a^ | not serious | not serious | none | 466 | 474 | - | MD **0.26 lower** (0.39 lower to 0.12 lower) | ⨁⨁⨁◯ MODERATE | IMPORTANT |
| Change in axial length from baseline (1 year) | | | | | | | | | | | | |
| 2 | randomised trials | not serious | not serious | not serious | serious ^b^ | none | 107 | 104 | - | MD **0.06 lower** (0.12 lower to 0 ) | ⨁⨁⨁◯ MODERATE | NOT IMPORTANT |
| Change in axial length from baseline (2 years) | | | | | | | | | | | | |
| 3 | randomised trials | not serious | serious ^c^ | not serious | serious ^d^ | none | 392 | 399 | - | MD **0.1 lower** (0.2 lower to 0 ) | ⨁⨁◯◯ LOW | IMPORTANT |
| Change in refractive error from baseline (1 year) | | | | | | | | | | | | |
| 2 | randomised trials | not serious | not serious | not serious | serious ^b^ | none | 102 | 104 | - | MD **0.1 lower** (0.21 lower to 0 ) | ⨁⨁⨁◯ MODERATE | NOT IMPORTANT |

**CI:** Confidence interval; **MD:** Mean difference

**Explanations**

a. I^2^ in the moderate range

b. CI overlaps no effect with small sample size (<400 participants)

c. Important, high I^2^

d. CI overlaps no effect

**Table S1. Handling of heterogeneity & meta-biases.**

| **Meta-analyses** | **Statistical software** | **Fixed/ Random Effects model** | **I^2^ or Cochran’s χ^2 /^Q or τ^2^ test** | **Funnel plot** | **Additional analysis** | **Risk of bias assessment** | **Selective outcome reporting** | **Dual co-authorship** | **Other sources of bias** |
| --- | --- | --- | --- | --- | --- | --- | --- | --- | --- |
| **Walline et al. 2011** | RevMan 5 | Fixed and random effects | -I^2^  -χ^2^ | No | -Sensitivity analysis  - Subgroup analysis | Cochrane Collaboration Risk of Bias Tool | Unlikely | Yes | Performance, attrition biases |
| **Sherwin et al. 2012** | Stata 10.1 | Random effects | -Cochran’s  -I^2^ | Some suggestion of asymmetry, Begg test, Egger test | - Sensitivity analysis  - Subgroup analysis | Tool proposed by Sanderson et al. 2007 | Possible | No | Reporting bias |
| **Song et al. 2011** | NA | Fixed and random effects | -Cochran’s  -τ^2^ | Symmetrical | - Egger test  - Random - effect meta -regression  - Sensitivity analysis | Jadad score ≥ 2 | Unlikely | No | External validity |
| **Li et al. 2011** | RevMan 5 | Fixed and random effects | -I^2^  -χ^2^ | Symmetrical | -Sensitivity analysis  -Subgroup analysis | Jadad score ≥ 3 | Unlikely | No | External validity |
| **Li et al. 2014** | RevMan 5.1 | Fixed and random effects | -I^2^  -χ^2^ | No | -Mann Whitney U test | Jadad score, Newcastle-Ottawa scale | Unlikely | No | External validity |
| **Sun et al. 2015** | Stata 11.0 | Fixed and random effects | -I^2^  -χ^2^ | No | -Sensitivity analysis  -Subgroup analysis | Jadad score, MINORS, GRADE | Unlikely | No | Selection bias |
| **Si et al. 2015** | RevMan 5.2.6 | Random effects | -I^2^  -χ^2^  -τ^2^ | No | -Sensitivity analysis | Jadad score | Unlikely | No | Possible selection bias |
| **Wen et al. 2015** | Stata 10.1 | Fixed and random effects | -I^2^  -χ^2^ | No | -Sensitivity analysis  -Subgroup analysis | Jadad score ≥ 4, Newcastle-Ottawa scale ≥ 6 | Unlikely | No | Small number of trials and sample sizes |
| **Li et al. 2016** | RevMan 5.3 | Fixed and random effects | -I^2^  -χ^2^ | No | -Subgroup analysis | Jadad score, Newcastle-Ottawa scale | Unlikely | No | Possible selection bias |
| **Li et al. 2017** | RevMan 5.3 | Fixed and random effects | -I^2^  -χ^2^ | No | -Subgroup analysis | Jadad score ≥ 4, Newcastle-Ottawa scale | Unlikely | No | External validity |
| (continued) | | | | | | | | | |
| **Huang et al. 2016** | Stata 10.0  WinBUGS 1.4 | Random effects | -I^2^ | No | -Sensitivity analysis  -‘Node splitting’  -Subgroup analysis | Cochrane Collaboration Risk of Bias Tool | Unlikely | No | Performance bias, small number of trials |
| **Xiong et al. 2017** | Stata 12.0 | Random effects | -I^2^  -τ^2^ | No | -Sensitivity analysis  -Subgroup analysis | Adapted checklist proposed by Downs and Black 1998^23^, score ≥ 60% | Unlikely | No | Possible selection and performance biases, bias due to numeric conversions |
| **Cui et al. 2017** | Comprehensive Meta-analysis software 2.0 | Random effects | -Cochran’s  -I^2^ | No | -Sensitivity analysis | Cochrane Collaboration Risk of Bias Tool | Unlikely | No | Small number of trials |
| **Gong et al. 2017** | RevMan 5.3,  Stata 12.0, SAS 9.4 | Random effects | -Q  -H  -I^2^ | Begg test, Egger test, trim-and-fill method | -Sensitivity analysis  -Subgroup analysis | Cochrane Collaboration 6 aspects of Bias tool, Newcastle-Ottawa scale ≥ 5 | Unlikely | No | External validity |

NA = not available; RevMan = Reference Manager

**Table S2. Unit of analysis for RCTs included in the meta-analysis.**

| **Unit of analysis** | **Unit of analysis used by primary studies included in the meta-analysis** |
| --- | --- |
| **Right eye, only** | Berntsen 2012; Charm 2013; Cheng 2010; Cho 2012; Edwards 2002; Katz 2003; Lam 2014; Shih 2001; Walline 2004; Yen 1989; Yi 2015 |
| **Right and left eye, separately** | Chan 2014; Parssinen 1989; Jensen 1991 |
| **Eye, randomised** | Chua 2006 |
| **Average of both eyes** | Adler 2006; Aller 2016; Bartlett 2003; Chung 2002; Fujikado 2014; Tan 2005 |
| **Average of both eyes or one eye only** | Anstice 2011, Gwiazda 2003 |
| **Not stated** | Cheng 2016; Paune 2015; Siatkowksi 2008; Yang 2009 |

RCTs, randomized controlled trials

**Table S3. Excluded studies after full text review.**

| **Selected for review** | **Reason for exclusion** |
| --- | --- |
| Smith MJ, Walline JJ, et al. Controlling myopia progression in children and adolescents. Adolesc Health Med Ther [Internet]. Dove Medical Press Ltd 2015; 6:133–40. | Non-systematic review |
| Van Meter WS, Musch DC, Jacobs DS, Kaufman SC, Reinhart WJ, Udell IJ, et al. Safety of overnight orthokeratology for myopia: a report by the American Academy of Ophthalmology. Ophthalmology. Elsevier Inc. 2008;115(12):2301. | Non-systematic review |
| Li X, Friedman IB, Medow NB, Zhang C. Update on Orthokeratology in Managing Progressive Myopia in Children: Efficacy, Mechanisms, and Concerns. J Pediatr Ophthalmol Strabismus; 1–7. | Non-systematic review |
| Xie P, Guo X, P. X, X. G. Chinese experiences on orthokeratology. Eye Contact Lens [Internet]. Lippincott Williams and Wilkins 2016;42(1):43–7. | Non-systematic review |
| Liu YM, Xie P. The Safety of Orthokeratology-A Systematic Review. Eye Contact Lens 2016;42(1):35–42. | Low quality of evidence |
| Kam, K. W., Yung, W., Li, G. K. H., Chen, L. J. & Young, A. L. Infectious keratitis and orthokeratology lens use: a systematic review. *Infection* 1–9 (2017). | Low quality of evidence |
| Pineles, S. L. *et al.* Atropine for the Prevention of Myopia Progression in Children: A Report by the American Academy of Ophthalmology. *Ophthalmology* (2017). | Contains low quality of evidence |
| Donovan L, Sankaridurg P, Ho A, Naduvilath T, Smith EL, A. Holden B, et al. Myopia progression rates in urban children wearing single-vision spectacles. Optom Vis Sci. 2012 Jan;89(1):27–32. | Intervention not in the scope of this overview |
| Pan C-W, Qian D-J, Saw S-M. Time outdoors, blood vitamin D status and myopia: a review. Photochem Photobiol Sci. Royal Society of Chemistry; 2017;16(3):426–32. | Topic not directly relevant |

|  | Table S4. Citation matrix | | | | | | | | | | | | | | | | | | | |
| --- | --- | --- | --- | --- | --- | --- | --- | --- | --- | --- | --- | --- | --- | --- | --- | --- | --- | --- | --- | --- |
| Systematic Reviews | | | | | | | | | | | | | | | | | | | | |
| Primary study | **Type of primary study** | **Cui 2017**^26^ | **Gong 2017**^12^ | **Li 2017**^14^ | **Xiong 2017**^15^ | **Huang 2016**^25^ | **Li 2016**^13^ | **Shih 2016**^19^ | **Chassine 2015**^29^ | **Si 2015**^23^ | **Sun 2015**^22^ | **Wen 2015**^24^ | **Li 2014**^28^ | **Sherwin 2012**^27^ | **Li 2011**^21^ | **Song 2011**^20^ | **Walline 2011**^8^ | **Wei 2011**^18^ | **Saw 2002**^9^ | **Total number of reviews (/18)** |
| Adler 2006 | RCT | - | - | - | - | **+** | - | - | - | - | - | - | - | - | - | - | **+** | - | - | **2** |
| Aller 2006 | RCT | - | - | - | - | **+** | - | - | - | - | - | - | - | - | - | - | **+** | - | - | **2** |
| Aller 2016 | RCT | - | - | **+** | - | - | - | - | - | - | - | - | - | - | - | - | - | - | - | **1** |
| Anstice 2011 | RCT | - | - | **+** | - | **+** | - | - | **+** | - | - | - | - | - | - | - | - | - | - | **2** |
| Bartlett 2003 | RCT | - | **-** | - | - | - | - | - | - | - | - | - | - | **+** | - | **-** | - | **-** | - | **1** |
| Berntsen 2012 | RCT | - | - | - | - | **+** | - | - | - | - | - | - | - | - | - | - | - | - | - | **1** |
| Chan 2014 | RCT | - | - | - | - | - | **+** | - | - | - | - | - | - | - | - | - | - | - | - | **1** |
| Charm 2013 | RCT | - | - | - | - | **+** | **+** | - | - | **+** | **+** | **+** | - | - | - | - | - | - | - | **5** |
| Cheng 2010 | RCT | - | - | - | - | - | - | - | - | - | - | - | - | - | **+** | - | **+** | - | - | **2** |
| Cheng 2014 | RCT | - | - | - | - | **+** | - | - | **+** | - | - | - | - | - | - | - | - | - | - | **2** |
| Cheng 2016 | RCT | - | - | **+** | - | - | - | - | - | - | - | - | - | - | - | - | - | - | - | **1** |
| Chia 2012 | RCT | - | **+** | - | - | **+** | - | **+** | **+** | - | - | - | - | - | - | - | - | - | - | **4** |
| Chia 2014 | RCT | - | - | - | - | - | - | - | **+** | - | - | - | - | - | - | - | - | - | - | **1** |
| Cho and Cheung 2012 | RCT | - | - | - | - | **+** | **+** | - | - | **+** | **+** | **+** | - | - | - | - | - | - | - | **5** |
| Chua 2006 | RCT | - | **+** | - | - | **+** | - | **+** | **+** | - | - | - | **+** | - | - | **+** | **+** | - | - | **7** |
| Chung 2002 | RCT | - | - | - | - | **+** | - | - | **+** | - | - | - | - | - | - | - | **+** | - | - | **3** |
| Edwards 2002 | RCT | - | - | - | - | **+** | - | - | - | - | - | - | - | - | **+** | - | **+** | - | - | **3** |
| Fujikado 2014 | RCT | - | - | **+** | - | - | - | - | - | - | - | - | - | - | - | - | - | - | - | **1** |
| Fulk 1996 | RCT | - | - | - | - | - | - | - | - | - | - | - | - | - | - | - | **+** | - | **+** | **2** |
| Fulk 2000 | RCT | - | - | - | - | **+** | - | - | - | - | - | - | - | - | **+** | - | - | - | **+** | **3** |
| Fulk 2002 | RCT | - | - | - | - | - | - | - | - | - | - | - | - | - | - | - | **+** | - | - | **1** |
| Grosvenor 1987 | RCT | - | - | - | - | - | - | - | - | - | - | - | - | - | - | - | **+** | - | **+** | **2** |
| Gwiazda 2003 | RCT | - | - | - | - | **+** | - | - | **+** | - | - | - | - | - | **+** | - | **+** | - | - | **4** |
| Hasebe 2008 | RCT | - | - | - | - | **+** | - | - | - | - | - | - | - | - | **+** | - | **+** | - | - | **3** |
| He 2015 | RCT | - | - | - | **+** | - | - | - | - | - | - | - | - | - | - | - | - | - | - | **1** |
| Horner 1999 | RCT | - | - | - | - | **+** | - | - | **+** | - | - | - | - | - | - | - | - | - | **+** | **3** |
| Hsiao 2005 | RCT | - | **+** | - | - | - | - | - | - | - | - | - | - | - | - | **+** | **+** | - | - | **3** |
| Jensen 1991 | RCT | - | - | - | - | **+** | - | - | - | - | - | - | - | - | - | - | **+** | - | **+** | **3** |
| JIn 2015 | CCT | - | - | - | **+** | - | - | - | - | - | - | - | - | - | - | - | - | - | - | **1** |
| Kang 2011 | RCT | **+** | - | - | - | - | - | - | **-** | - | - | - | - | - | - | - | - | - | - | **1** |
| Katz 2003 | RCT | **+** | - | - | - | **+** | - | - | - | - | - | - | - | - | - | - | **+** | - | - | **3** |
| Khoo 1999 | RCT | **+** | - | - | **-** | - | - | - | - | - | - | - | - | - | - | - | - | - | - | **1** |
| Kumaran 2015 | RCT | - | **+** | - | - | - | - | - | - | - | - | - | - | - | - | - | - | - | - | **1** |
| Lam 2014 | RCT | - | - | **+** | - | **+** | - | - | **+** | - | - | - | - | - | - | - | - | - | - | **3** |
| Leung 1999 | RCT | - | - | - | - | **+** | - | - | **+** | - | - | - | - | - | **+** | - | - | - | - | **3** |
| Liang 2008 | RCT | - | **+** | - | - | - | - | - | - | - | - | - | - | - | - | **+** | - | **+** | - | **3** |
| Parssinen 1989 | RCT | - | - | - | - | **+** | - | - | - | - | - | - | - | - | **+** | - | **+** | - | **+** | **4** |
| Paune 2015 | CCT | - | - | **+** | - | - | - | - | - | - | - | - | - | - | - | - | - | - | - | **1** |
| Sankaridurg 2010 | RCT | - | - | - | - | **+** | - | - | - | - | - | - | - | - | - | - | **+** | - | - | **2** |
| Schwartz 1981 | RCT | - | - | - | - | - | - | - | - | - | - | - | - | - | - | - | **+** | - | **+** | **2** |
| Shih 1999 | RCT | - | **+** | - | - | **+** | - | **+** | - | - | - | - | **+** | - | - | **+** | **+** | - | **+** | **7** |
| Shih 2000 | RCT | - | - | - | - | - | - | - | - | - | - | - | - | - | - | - | - | - | **+** | **1** |
| Shih 2001 | RCT | - | **+** | - | - | **+** | - | **+** | - | - | - | - | **+** | - | **+** | - | - | - | - | **5** |
| Siatkowski 2008 | RCT | - | - | - | - | **+** | - | - | - | - | - | - | - | - | - | - | **+** | - | - | **2** |
| Swarbrick 2015 | RCT | **+** | - | - | - | - | - | - | **+** | - | - | - | - | - | - | - | - | - | - | **2** |
| Tan 2005 | RCT | - | - | - | - | **+** | - | - | - | - | - | - | - | - | - | - | **+** | - | - | **2** |
| Walline 2004 | RCT | **+** | - | - | - | **+** | - | - | **+** | - | - | - | - | - | - | - | **+** | - | - | **4** |
| Walline 2008 | RCT | - | - | - | - | **+** | - | - | - | - | - | - | - | - | - | - | - | - | - | **1** |
| Wu 2013 | CCT | - | - | - | **+** | - | - | - | - | - | - | - | - | - | - | - | - | - | - | **1** |
| Yang 2009 | RCT | - | - | - | - | **+** | - | - | - | - | - | - | - | - | **+** | - | **+** | - | - | **3** |
| Yeh 2008 | RCT | - | - | - | - | - | - | - | - | - | - | - | - | - | - | - | - | **+** | - | **1** |
| Yen 1989 | RCT | - | **+** | - | - | **+** | - | **+** | - | - | - | - | **+** | - | - | **+** | **+** | - | **+** | **7** |
| Yi 2015 | RCT | - | **+** | - | - | - | - | - | - | - | - | - | - | - | - | - | - | - | - | **1** |
| Yi & Lee 2011 | RCT | - | - | - | **+** | **+** | - | - | - | - | - | - | - | **+** | - | - | - | - | - | **3** |
| Blacker 2009 | cohort | - | - | - | - | - | - | - | **+** | - | - | - | - | - | - | - | - | - | - | **1** |
| Brodstein 1984 | cohort | - | **+** | - | - | - | - | - | - | - | - | - | **+** | - | - | - | - | - | - | **2** |
| Chen 2013 | cohort | - | - | - | - | - | **+** | - | **+** | **+** | **+** | **+** | - | - | - | - | - | - | - | **5** |
| Cho 2005 | cohort | - | - | - | - | - | **+** | - | - | **+** | **+** | **+** | - | - | - | - | - | - | - | **4** |
| Chou 1997 | cohort | - | **+** | - | - | - | - | - | - | - | - | - | **+** | - | - | - | - | - | - | **2** |
| Chua 2015 | cohort | - | - | - | **+** | - | - | - | - | - | - | - | - | - | - | - | - | - | - | **1** |
| Clark and Clark 2015 | cohort | - | **+** | - | - | - | - | - | - | - | - | - | - | - | - | - | - | - | - | **1** |
| Deng 2010 | cohort | - | - | - | **+** | - | - | - | - | - | - | - | - | - | - | - | - | - | - | **1** |
| Dirani 2009 | cohort | - | - | - | **+** | - | - | - | **+** | - | - | - | - | **+** | - | - | - | - | - | **3** |
| Fan 2007 | cohort | - | **+** | - | - | - | - | - | - | - | - | - | **+** | - | - | **+** | - | - | - | **3** |
| Fang 2010 | cohort | - | **+** | - | - | - | - | - | - | - | - | - | **+** | - | - | - | - | - | - | **2** |
| French 2013 | cohort | - | - | - | **+** | - | - | - | - | - | - | - | - | - | - | - | - | - | - | **1** |
| Guggenheim 2012 | cohort | - | - | - | **+** | - | - | - | - | - | - | - | - | - | - | - | - | - | - | **1** |
| Guo 2013 | cohort | - | - | - | **+** | - | - | - | **+** | - | - | - | - | - | - | - | - | - | - | **2** |
| Guo 2015 | cohort | - | - | - | **+** | - | - | - | - | - | - | - | - | - | - | - | - | - | - | **1** |
| Hiraoka 2012 | cohort | - | - | - | - | - | **+** | - | - | - | - | **+** | - | - | - | - | - | - | - | **2** |
| Ip 2008 | cohort | - | - | - | **+** | - | - | - | - | - | - | - | - | **+** | - | - | - | - | - | **2** |
| Ip 2008 (2) | cohort | - | - | - | - | - | - | - | - | - | - | - | - | **+** | - | - | - | - | - | **1** |
| Jones 2007 | cohort | - | - | - | **+** | - | - | - | - | - | - | - | - | **+** | - | - | - | - | - | **2** |
| Jones - Jordan 2011 | cohort | - | - | - | **+** | - | - | - | - | - | - | - | - | **+** | - | - | - | - | - | **2** |
| Kakita 2011 | cohort | - | - | - | - | - | **+** | - | - | **+** | **+** | **+** | - | - | - | - | - | - | - | **4** |
| Kennedy 2000 | cohort | - | **+** | - | - | - | - | - | - | - | - | - | **+** | - | - | - | - | - | - | **2** |
| Khader 2006 | cohort | - | - | - |  | - | - | - | - | - | - | - | - | **+** | - | - | - | - | - | **1** |
| Lee 2006 | cohort | - | **+** | - | - | - | - | - | - | - | - | - | **+** | - | - | - | - | - | - | **2** |
| Lee 2015 | cohort | - | - | - | **+** | - | - | - | - | - | - | - | - | - | - | - | - | - | - | **1** |
| Li 2015 | cohort | - | - | - | **+** | - | - | - | - | - | - | - | - | - | - | - | - | - | - | **1** |
| Lin 2013 | cohort | - | **+** | - | - | - | - | - | - | - | - | - | - | - | - | - | - | - | - | **1** |
| Lin 2014 | cohort | - | **+** | - | - | - | - | - | **+** | - | - | - | - | - | - | - | - | - | - | **2** |
| Low 2010 | cohort | - | - | - | **+** | - | - | - | - | - | - | - | - | **+** | - | - | - | - | - | **2** |
| Lu 2009 | cohort | - | - | - | **+** | - | - | - | - | - | - | - | - | **+** | - | - | - | - | - | **2** |
| Ma 2010 | cohort | - | - | - | - | - | - | - | - | - | - | - | - | **+** | - | - | - | - | - | **1** |
| Mutti 2002 | cohort | - | - | - | **+** | - | - | - | - | - | - | - | - | **+** | - | - | - | - | - | **2** |
| Onal 2007 | cohort | - | - | - | - | - | - | - | - | - | - | - | - | **+** | - | - | - | - | - | **1** |
| Oner 2015 | cohort | - | - | - | **+** | - | - | - | - | - | - | - | - | - | - | - | - | - | - | **1** |
| Pan 2015 | cohort | - | - | - | **+** | - | - | - | - | - | - | - | - | - | - | - | - | - | - | **1** |
| Parssinen & Lyra 1993 | cohort | - | - | - | - | - | - | - | - | - | - | - | - | **+** | - | - | - | - | - | **1** |
| Peckham 1977 | cohort | - | - | - | - | - | - | - | - | - | - | - | - | **+** | - | - | - | - | - | **1** |
| Rose 2008 | cohort | - | - | - | - | - | - | - | **+** | - | - | - | - | **+** | - | - | - | - | - | **2** |
| Rose 2008 (2) | cohort | - | - | - | - | - | - | - | - | - | - | - | - | **+** | - | - | - | - | - | **1** |
| Sankaridurg 2011 | cohort | - | - | **+** | - | - | - | - | - | - | - | - | - | - | - | - | - | - | - | **1** |
| Santodomingo - Rubido 2012 | cohort | - | - | - | - | - | **+** | - | - | **+** | **+** | **+** | - | - | - | - | - | - | - | **4** |
| Saw 2000 | cohort | - | - | - | **+** | - | - | - | - | - | - | - | - | **+** | - | - | - | - | - | **2** |
| Saw 2001 | cohort | - | - | - | - | - | - | - | - | - | - | - | - | **+** | - | - | - | - | - | **1** |
| Saw 2002 | cohort | - | - | - | - | - | - | - | - | - | - | - | - | **+** | - | - | - | - | - | **1** |
| Saw 2006 | cohort | - | - | - | **+** | - | - | - | - | - | - | - | - | **+** | - | - | - | - | - | **2** |
| Tan 2000 | cohort | - | - | - | - | - | - | - | - | - | - | - | - | **+** | - | - | - | - | - | **1** |
| Vasudevan 2014 | cohort | - | - | - | - | - | - | - | **+** | - | - | - | - | - | - | - | - | - | - | **1** |
| Walline 2009 | cohort | - | - | - | - | - | - | - | - | **+** | **+** | **+** | - | - | - | - | - | - | - | **3** |
| Walline 2013 | cohort | - | - | **+** | - | - | - | - | - | - | - | - | - | - | - | - | - | - | - | **1** |
| Wu 2010 | cohort | - | - | - | - | - | - | - | - | - | - | - | - | **+** | - | - | - | - | - | **1** |
| Wu 2011 | cohort | - | **+** | - | - | - | - | - | - | - | - | - | **+** | - | - | - | - | - | - | **2** |
| Zhang 2010 | cohort | - | - | - | - | - | - | - | - | - | - | - | - | **+** | - | - | - | - | - | **1** |
| Zhou 2014 | cohort | - | - | - | **+** | - | - | - | - | - | - | - | - | - | - | - | - | - | - | **1** |
| Zhou 2015 | cohort | - | - | - | **+** | - | - | - | - | - | - | - | - | - | - | - | - | - | - | **1** |
| Zhu 2014 | cohort | - | - | - | - | - | **+** | - | - | - | - | - | - | - | - | - | - | - | - | **1** |

**e-Table 5. Citation Matrix**. Systematic reviews are represented in columns and primary studies in rows. (+) indicates primary studies contained in a systematic review and (-) studies not

included in a systematic review.

**Table S5. Methodological quality of included systematic reviews and meta-analyses based on ROBIS tool.**

| **Review and year** | **ROBIS Assessment** | | | | |
| --- | --- | --- | --- | --- | --- |
|  | **Domain 1: Study eligibility criteria** | **Domain 2: Identification**  **and selection**  **of studies** | **Domain 3: Data collection and study appraisal** | **Domain 4: Synthesis and findings** | **Risk of bias** |
| Cui 2017 | Low | Low | Low | Low | Low |
| Gong 2017 | Low | Low | Low | Low | Low |
| Li 2017 | Low | Low | Low | Low | Low |
| Xiong 2017 | Low | Low | Low | Unclear | Unclear |
| Huang 2016 | Low | Low | Low | Low | Low |
| Li 2016 | Low | Low | Low | Low | Low |
| Shih 2016 | Low | Low | Unclear | Low | Low |
| Chassine 2015 | High | Unclear | High | High | High |
| Si 2015 | Low | Low | Low | Low | Low |
| Sun 2015 | Low | Low | Low | Low | Low |
| Wen 2015 | Low | Low | Low | Low | Low |
| Li 2014 | Low | Low | Low | High | Unclear |
| Sherwin 2012 | Low | Low | Low | High | Unclear |
| Li 2011 | Low | Low | Low | Low | Low |
| Song 2011 | Low | Unclear | Low | Low | Low |
| Walline 2011 | Low | Low | Low | Low | Low |
| Wei 2011 | Low | Low | Low | Low | Low |
| Saw 2002 | High | Low | Unclear | Low | Low |

**Table S6. Methodological quality of included RCTs assessed using the Cochrane Collaboration Risk of Bias tool.**

**
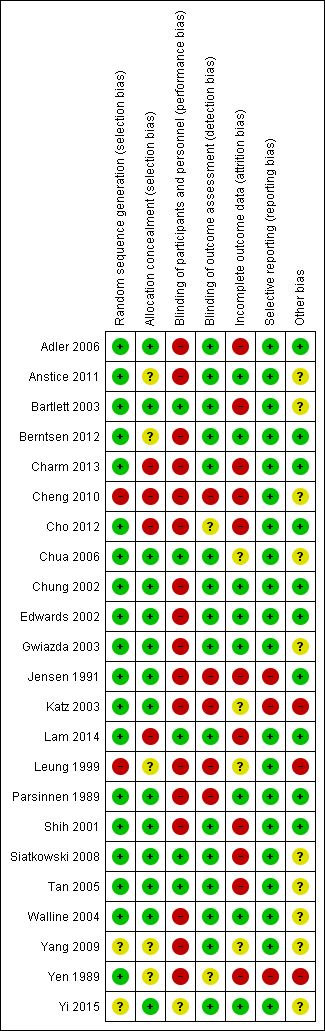
**

+, low risk of bias; ?, unclear risk of bias; -, high risk of bias; RCTs, randomized controlled trial

**Table S7. Methodological quality of included RCTs assessed using Jadad Scale.**

| **Primary study** | **Randomisation** | **Blinding** | **Lost to follow up** | **Allocation concealment** | **Analysis method** | **Jadad score** |
| --- | --- | --- | --- | --- | --- | --- |
| Aller 2016 | Adequate | DB | Adequate | Adequate | ITT | 5 |
| Chan 2014 | Unclear | NO | Adequate | NO | ITT | 2 |
| Cheng 2016 | Adequate | DB | Adequate | Adequate | ITT | 5 |
| Fujikado 2014 | Adequate | SB | Adequate | Adequate | PP | 4 |

DB, double binding; SB, single blinding; NO, no blinding or no allocation concealment; PP, per-protocol analysis; ITT, intention-to-treat analysis; RCTs, randomized controlled trials

**Table S8. Methodological quality of included cohort studies assessed using Newcastle-Ottawa Quality Assessment Scale.**

|  | **Selection** | | | |  | **Outcome** | | | |
| --- | --- | --- | --- | --- | --- | --- | --- | --- | --- |
| **Primary study** | **Exposed cohort representative** | **Non-exposed cohort selection** | **Exposure ascertainment** | **Outcome not present at start** | **Comparability of cohorts** | **Assessment** | **Follow-up length** | **Follow-up adequacy** | **NOS score** |
| Brodstein 1984 | * | * | * | * | * | * | * | * | 8 |
| Chen 2013 | * | * | * | * | ** | * | * | * | 9 |
| Cho 2005 | * | * | * | * | ** | * | * | * | 9 |
| Clark 2015 | * | * | * |  | ** | * | * | * | 7 |
| Fan 2007 | * | * | * | * | ** | * | * | * | 9 |
| Fang 2010 | * | * | * | * | ** | * | * | * | 9 |
| Hiraoka 2012 | * | * | * | * | * | * | * | * | 8 |
| Kakita 2011 | * | * | * | * | * | * | * | * | 8 |
| Kennedy 2000 | * | * | * | * | ** | * | * | * | 9 |
| Lee 2006 | * | * | * | * | ** | * | * | * | 9 |
| Paune 2015 | * | * | * | * | ** | * | * | * | 9 |
| Sankaridurg 2011 | * | * | * | * | ** | * | * | * | 9 |
| Santodomingo – Rubido 2012 | * | * | * | * | ** | * | * | * | 9 |
| Walline 2009 | * | * | * | * | ** | * | * | * | 9 |
| Walline 2013 | * | * | * | * | ** | * | * | * | 9 |
| Wu 2011 | * | * | * | * | ** | * | * | * | 9 |
| Zhu 2014 | * | * | * |  | ** | * | * | * | 8 |

*indicates score. A study can be awarded a maximum of one star for each item in the Selection and Outcome categories. A maximum of two stars can be awarded for comparability.

**Table S9. Summary of qualitative and quantitative findings of each study.**

| **Review** | **Results** | **Review Limitations** | **Conclusions** |
| --- | --- | --- | --- |
| **Walline et al. 2011** | Anti-muscarinic agents and multifocals appear effective, RGPCLs show no evidence of effect, undercorrection increases myopia progression. | Protocol amendment, several included funded studies, no blinding in many included studies, possible attrition bias – only 3 studies performed ITT, funded review, dual co-authorship | Anti-muscarinic topical medication appears as the most effective intervention, despite side effects. Further investigation of corneal reshaping contact lenses or soft bifocal contact lenses is warranted. |
| **Sherwin et al. 2012** | Increase in hours per week of time spent outdoors associated with reduced odds of myopia. | Conference abstracts excluded,  variation between studies, differences in outdoor exposure definitions, more excluded studies from meta-analysis than included, overlapping, possible publication and reporting biases | Protective association between increasing time spent outdoors and prevalent myopia. |
| **Saw et al. 2002** | Inconclusive. Some evidence of 0.5% atropine eyedrops efficacy in slowing myopia progression. | Majority of included RCTs performed per protocol analysis, potential exclusion of relevant studies, various follow-up periods | Some evidence of 0.5% atropine eyedrops efficacy in slowing myopia progression. Larger RCTs with longer follow-up periods, as well as studies investigating pirenzepine needed. |
| **Wei et al. 2011** | No difference in AL between atropine + acupuncture and atropine only groups. | Only 2 trials included, analysis based on the mean of the two eyes change of refractive error, possible selection bias in 1 RCT, possible selective outcome reporting, non-English trials may have not been identified | No conclusions can be drawn for the benefit of acupuncture for slowing myopia progression. No recommendation for clinical use can be made until further research is conducted. |
| **Chassine et al. 2015** | Atropine, OK and bifocal devices can slow myopia progression. Time spent outdoors is important in preventing myopia. | Language limitation, RCTs and observational included studies, possible selective outcome reporting | Atropine, OK and bifocal devices can slow myopia progression. Further research and larger studies needed. |
| **Wen et al. 2015** | OK demonstrates efficacy and acceptability on myopic control, which appears to decrease with increasing age. | Small sample size of included studies, dioptric refractive error and age not analyzed, only 2 year follow-up with exclusion of 1 study | OK is effective and acceptable in restricting myopic progression with careful education and monitoring. |
| **Xiong et al. 2017** | Outdoor exposure has a protective effect on the onset of myopia, but not on myopia progression in already myopic individuals. | Heterogeneity due to different study designs/age/ethnicities, possible noncompliance with control in clinical trials, possible overestimation of effect due to implemented method of assessment, bias due to conversions of original estimates | Protective effect of improved outdoor time for onset but not progression of myopia. |
| **Gong et al. 2017** | Atropine slows myopia progression. | Different types of studies combined, incomprehensive reports of adverse events, possible rebound effect and poor near vision acuity after atropine were not investigated, AL could not be evaluated | Atropine shows a dose independent efficacy in restriction of myopic progression. Adverse events are dose dependent. Production of 0.01% atropine commercially could aid future research. |
| **Shih et al. 2016** | Atropine slows myopia progression. | Language restriction, only published studies included | Daily treatment with 0.01% atropine solution appears to provide the most effective and safe profile. |
| **Song et al. 2011** | Atropine slows myopia progression. | 1 year follow-up, only 2 studies reported on AL elongation | Positive dose -response relation for atropine and myopic change. |
| **Li et al. 2011** | Multifocal lenses slow myopia progression by 0.25D/year compared to SVLs and show greater effect in higher myopia at baseline and Asians. | Various follow-up periods, only published studies included | Multifocal lenses appear more efficient in slowing myopia progression in schoolchildren compared to SVLs. |
| **Li et al. 2014** | Atropine slows myopia progression in Asian and white populations. | Different follow-up periods, separate evaluation of atropine effect in RCTS and cohorts, various ethnicities, various atropine concentrations, language restriction, external validity compromised | Atropine slows myopia progression more in Asian than white populations  Slowing of myopia progression independent of atropine concentration. |
| **Sun et al. 2015** | Myopia progression is slower with OK compared to spectacles or soft contact lenses. | Small sample size, selection bias, only 2 studies report on VCD | OK slows myopia progression. |
| **Si et al. 2015** | Myopia progression is slower with OK compared to SVLs or soft contact lenses. | Non-published conference abstracts excluded, various ethnicities, small sample size, only 2 studies report on VCD | OK slows myopia progression more in Asian than Caucasian populations. |
| **Li et al. 2016** | Myopia progression is slower with OK compared to spectacles. | Language restriction, mainly Asian populations, only AL assessed | OK slows myopia progression. |
| **Li et al. 2017** | Concentric ring bifocal and peripheral add multifocal SCLs are both effective in controlling myopia progression. Concentric ring bifocal lenses appear more effective. | Various follow-up periods, majority of studies in white children | SCLs with concentric ring bifocal and peripheral add multifocal designs can slow myopia progression by 0.2-0.3D/year and decrease axial elongation by ~0.10mm/year, with treatment effect lasting for 24 months or longer. |
| **Huang et al. 2016** | Atropine is superior to all interventions. Pirenzepine, OK, peripheral defocus modifying CLs, cyclopentolate, prismatic bifocals have moderate effects, PALs, bifocals, peripheral defocus modifying spectacles and outdoor activities have weak effects in controlling myopia progression. RGPCLs, soft CLs, undercorrection, timolol are ineffective.  Asian children benefit more and interventions lose their early effect during the second year. | Variable interventions, both placebo and single vision spectacles used as control, variable quality of RCTs, performance bias of RCTs  No information on safety, small trial sizes | RGPCLs, soft CLs, undercorrection, timolol are ineffective in myopia control. Atropine, pirenzepine, OK, peripheral defocus modifying CLs, PALs produce statistically significant reduction in myopia progression. Side effects of atropine, cost, complexity of OK and limited efficacy of PALs constitute limitations. Atropine 0.01%, pirenzepine, peripheral defocus modifying CLs appear as viable options for slowing myopia progression. |

AL = axial length; CLs = contact lenses; NA = not available; OK = orthokeratology; PALs = progressive addition lenses; RGPCLs = rigid gas permeable contact lenses; SER = spherical equivalent refraction; SVLs = single vision lenses; VCD = vitreous chamber
